# Uterine macrophages and NK cells exhibit population and gene-level changes after implantation but maintain pro-invasive properties

**DOI:** 10.3389/fimmu.2024.1364036

**Published:** 2024-03-19

**Authors:** Sneha Mani, James Garifallou, Se-jeong Kim, Michael K. Simoni, Dan Dongeun Huh, Scott M. Gordon, Monica Mainigi

**Affiliations:** ^1^ Division of Reproductive Endocrinology and Infertility, University of Pennsylvania, Perelman School of Medicine, Philadelphia, PA, United States; ^2^ Division of Neonatology, Children’s Hospital of Philadelphia, Philadelphia, PA, United States; ^3^ Department of Bioengineering, School of Engineering and Applied Science, University of Pennsylvania, Philadelphia, PA, United States; ^4^ National Science Foundation (NSF) Science and Technology Center for Engineering Mechanobiology, University of Pennsylvania, Philadelphia, PA, United States; ^5^ Institute for Regenerative Medicine, Perelman School of Medicine, University of Pennsylvania, Philadelphia, PA, United States; ^6^ Department of Pediatrics, University of Pennsylvania, Perelman School of Medicine, Philadelphia, PA, United States

**Keywords:** placentation, uterine macrophages, uterine NK cells, organ-on-a-chip, endometrium, decidua, implantation

## Abstract

**Introduction:**

Prior to pregnancy, hormonal changes lead to cellular adaptations in the endometrium allowing for embryo implantation. Critical for successful pregnancy establishment, innate immune cells constitute a significant proportion of uterine cells prior to arrival of the embryo and throughout the first trimester in humans and animal models. Abnormal uterine immune cell function during implantation is believed to play a role in multiple adverse pregnancy outcomes. Current work in humans has focused on uterine immune cells present after pregnancy establishment, and limited in vitro models exist to explore unique functions of these cells.

**Methods:**

With single-cell RNA-sequencing (scRNAseq), we comprehensively compared the human uterine immune landscape of the endometrium during the window of implantation and the decidua during the first trimester of pregnancy.

**Results:**

We uncovered global and cell-type-specific gene signatures for each timepoint. Immune cells in the endometrium prior to implantation expressed genes associated with immune metabolism, division, and activation. In contrast, we observed widespread interferon signaling during the first trimester of pregnancy. We also provide evidence of specific inflammatory pathways enriched in pre- and post-implantation macrophages and natural killer (NK) cells in the uterine lining. Using our novel implantation-on-a-chip (IOC) to model human implantation ex vivo, we demonstrate for the first time that uterine macrophages strongly promote invasion of extravillous trophoblasts (EVTs), a process essential for pregnancy establishment. Pre- and post-implantation uterine macrophages promoted EVT invasion to a similar degree as pre- and post-implantation NK cells on the IOC.

**Conclusions:**

This work provides a foundation for further investigation of the individual roles of uterine immune cell subtypes present prior to embryo implantation and during early pregnancy, which will be critical for our understanding of pregnancy complications associated with abnormal trophoblast invasion and placentation.

## Introduction

Perinatal complications result in over 300,000 maternal deaths and 15 million premature deliveries worldwide every year (1). Miscarriage, preeclampsia, intrauterine fetal growth restriction, preterm birth, and placenta accreta, have all been associated with abnormalities in pregnancy establishment (2–7). A receptive maternal uterus is essential for successful embryo implantation and formation of the placenta, or placentation (8). Conversely, an abnormal pre-pregnancy maternal uterine environment, caused by pre-conception exposures or other factors, can increase the risk for adverse perinatal outcomes associated with disordered placentation (9–11). Characterizing the uterine cellular microenvironment and understanding how specific maternal uterine cells participate in normal pregnancy can therefore provide insight into the pathophysiology of several pregnancy complications.

In humans, the uterine lining, or endometrium, prepares for implantation prior to embryo arrival (12). After ovulation, during the mid-secretory phase of the menstrual cycle (*pre-implantation, mid-secretory endometrium, or MSE*), the endometrium remodels dramatically under hormonal influence to allow for a ‘window of implantation’, the period of endometrial receptivity that allows for embryo attachment and implantation. If implantation is successful, these cellular changes persist, and the portion of uterus into which fetal trophoblast cells invade and form the placenta is now known as the *post-implantation, first trimester (FT) decidua*. Examining changes across the MSE through the early first trimester of pregnancy is therefore required to capture an accurate understanding of how maternal cells support pregnancy establishment.

Immune cells are abundant in both MSE and FT decidua. Fluctuations in immune cell abundance and location coincide with processes critical to early pregnancy establishment (13–15). Further highlighting their involvement in establishing and maintaining pregnancy, abnormal number and function of uterine immune cells are associated with several adverse pregnancy outcomes (16, 17). In the pre- and early post-implantation uterine lining, natural killer (NK) cells and macrophages are the most abundant immune cells. Through the secretion of cytokines and growth factors in close proximity to invading fetal trophoblasts known as extravillous trophoblasts (EVTs), uterine NK (uNK) cells can regulate trophoblast invasion and are essential for remodeling of maternal uterine vessels (18–24). Data from *in vitro* studies have also suggested that decidual macrophages participate in tissue and vascular remodeling, trophoblast invasion, and clearance of apoptotic cells from the maternal-fetal interface (25–28).

To our knowledge, the majority of studies have focused on describing first trimester innate immune cells obtained from early pregnancy termination tissue, which does not account for their functions at the time of implantation (15). Limited studies suggest pre-implantation uNK cells differ transcriptionally and phenotypically from post-implantation uNK cells. Prior comparison of bulk endometrial NK cells from elective hysterectomies and bulk first trimester decidual NK cells showed gene expression differences across these cell types, both of which also differed from circulating peripheral NK cells (29). Another study found that pre-pregnancy NK cells possessed a unique repertoire of chemokine receptors, and were not cytotoxic until activated by IL-15, expressed robustly by decidualized endometrial stromal cells during the window of implantation (30, 31). Traditional assessments of NK cell function may not apply to uNK cells, as they are unique and distinct from peripheral NK cells.

Another limitation of several existing studies is assessment of bulk populations of immune cells based on surface markers identified in peripheral immune cell populations. Multiple subsets of immune cells exist based on transcriptome (32), surface phenotype by mass cytometry (33), secretome (20, 34, 35), and function *in vitro* (21). Several recent investigations have shed light on phenotype, ontogeny, function, and microanatomic location of uterine and fetal macrophages, but our understanding of specific roles for macrophages in early pregnancy establishment remains limited (36–40).

Given the need to comprehensively define and compare endometrial and decidual immune cells, we performed scRNAseq of CD45+ cells obtained from implantation window, mid-secretory phase endometrium (MSE) and the decidua from healthy first trimester (FT) pregnancies. We provide a sensitive and unbiased assessment of maternal immune cell subsets with as-yet-unexplored functions in early pregnancy. Given the predominance of the NK cell and macrophage compartments in the uterine lining during the peri-implantation period, we also assessed the ability of endometrial and decidual NK cells and macrophages to promote invasion of primary EVTs on an implantation-on-a-chip device (IOC) (41, 42) that we recently developed. Our data show that subpopulations of NK cells and macrophages are dynamic from the pre- to post-implantation period, changing both in composition and gene expression. We also provide evidence that bulk endometrial and decidual macrophages promote invasion of primary EVTs on the IOC to the same extent as bulk endometrial and decidual NK cells. These data lay the groundwork for further dissection of functions for individual innate immune subpopulations in normal and pathologic establishment and maintenance of early pregnancy.

## Materials and methods

### Human sample collection

All human tissue collection was approved by the IRB at the University of Pennsylvania. Endometrial biopsies (n=4) were obtained from women with regular menstrual cycles (between 25-35 days), with no significant medical history, not utilizing hormonal contraception or attempting pregnancy (IRB #82813). Biopsies were obtained from subjects 8 days after an LH surge, which was detected in the urine by the Clearblue^®^ Ovulation Test. Biopsies were obtained using a Pipelle^®^ Endometrial Suction Curette (CooperSurgical,Part # 8200). First trimester pregnancy tissue (n=4, gestational ages 5-12 weeks) was collected from the Penn Family Planning and Pregnancy Loss Center following elective pregnancy terminations with patient consent in accordance with current regulations regarding the use of human fetal tissue (IRB#827072). Patients with preexisting medical conditions or any pregnancy complications were excluded from the study. All collected tissue was kept on ice and cell isolation was carried out within 1 hour of obtaining tissue.

### Tissue processing

Endometrial and decidual tissues were grossly dissected and washed in PBS, then finely minced before being digested in a shaking 37°C water bath for 15-30 mins in sterile medium containing 0.28WU/mL Liberase TM (Roche, Catalog # 05401127001) and 30ug/mL DNase (Roche, catalog # 10104159001)]. A large-bore transfer pipet was used to pipet tissue up and down during digestion to break down tissue. Digested tissue was pelleted, subjected to RBC lysis, and then filtered through a 70μm filter.

For the fresh/unfrozen tissue, cells were processed within 2 hours for flow sorting. Frozen samples were similarly processed within 2 hours, and frozen in cryotubes at a concentration of 1x10^7^/mL in 1mL of freezing medium, consisting of 90% RPMI (ThermoFisher, Catalog # 11875085), 10% DMSO (Sigma, Catalog # D2650-100mL), and 1mg/mL BSA (Sigma, A9418)]. Samples were frozen in a Mr.Frosty freezing container at -80°C for 24 hours before being placed in a -120°C freezer for up to 6 months. Immediately prior to flow sorting, cryovials were thawed for 30 seconds in a 37°C water bath and immediately washed in PBS to remove DMSO.

### Flow sorting immune cells for scRNAseq and the IOC device

Fresh or cryopreserved single-cell suspensions from endometrial and decidual tissue, above, were stained with LIVE/DEAD Fixable Aqua Dead Cell Stain (Invitrogen, Cat# L34965) per manufacturer instructions. Cells were washed and treated with Human Fc Block (BD Biosciences, Cat# 564219, 1:200). For CD45+ cell purification for scRNAseq, cells were then washed and stained with CD45 (PerCP-Cy5.5, BioLegend, Clone 2D1, Cat# 368503, 1:100). All scRNAseq data presented, with the exception of **Supplementary Figure 3**, were generated from exclusively cryopreserved cells. For bulk NK (CD56+CD3-) and bulk macrophage (CD14+CD64+) cell purification for functional IOC experiments, cells were additionally stained with CD3 (BV785, BioLegend, Clone UCHT1, Cat# 300471, 1:100); CD14 (V450, BD Biosciences, Clone MφP9, Cat# 560349, 1:200); CD56 (PE, BD Biosciences, Clone B159, Cat# 555516, 1:50); and CD64 (PE-Cy7, BioLegend, Clone 10.1, Cat# 305022, 1:100). Antibody staining was performed at 4°C for 30 minutes in the dark. A BD FACSMelody sorter was used. All cells used for functional IOC experiments were isolated from cryopreserved single-cell suspensions. Cytospins were prepared in a cytocentrifuge at 600RPM for 3 minutes, after which cells were stained with hematoxylin and eosin.

### Single cell RNA sequencing

Next-generation sequencing libraries were prepared using the 10x Genomics Chromium Single Cell 3’ Reagent kit v3 as per manufacturer’s instructions. Libraries were uniquely indexed using the Chromium i7 Sample Index Kit, pooled, and sequenced on the Illumina NovaSeq6000 sequencer in a paired-end, single indexing run. Sequencing for each library targeted 20,000 mean reads per cell. Data was then processed using the Cellranger pipeline (10x genomics, v.6.0.0) for demultiplexing and alignment of sequencing reads to the GRCh38 transcriptome and creation of feature-barcode matrices.

Downstream analysis was performed using Seurat v4.0 (43) in the R statistical computing environment. Cells included in the analysis had between 200 and 4000 detected genes and a mitochondrial gene expression ratio of less than 20%. The dataset was split by condition (Endometrium and First Trimester), normalized using the sctransform v2 method while regressing out differences in mitochondrial content, and a principal component analysis was performed. Integration of the datasets across the two conditions was performed by utilizing the top variable gene features across the conditions and the FindIntegrationAnchors function. Downstream PCA, UMAP, and clustering with resolution of 0.9 were performed on the integrated dataset. Clustering resolution was adjusted to optimize biological variability expected based on prior studies and interrogation of resultant cluster distinguishing marker genes. P-values for differential gene expression testing were adjusted via the Bonferroni method for multiple testing correction. A gene was considered significantly differentially expressed (DEG) if 1) the value of the average log2 fold change was greater than 0.33 or less than -0.42 corresponding to a 25% increase or decrease in expression and 2) the adjusted p-value was less than 0.05.

### Implantation on a chip device experiment set up and cell isolation

Each experimental run consisted of five cellular combinations, and each device was run concurrently in triplicate: 1) co-culture of endothelial cells (ECs) and EVTs, 2) triculture of ECs, EVTs, and addition of 7000 uNK cells from the endometrium to the extracellular matrix (ECM), 3) triculture of ECs, EVTs, and addition of 7000 uNK cells isolated from first trimester decidua to the ECM, 4) triculture of ECs, EVTs, and addition of 7000 macrophages isolated from the endometrium to the extracellular matrix (ECM), and 5) triculture of ECs, EVTs, and addition of 7000 macrophages isolated from first trimester decidua to the ECM. In addition, three biological replicates were performed each with different human subject samples.

#### Extravillous trophoblast isolation and culture

Extravillous trophoblasts (EVTs) were collected from placental villi obtained from first trimester pregnancies as described above. Collected tissue was kept on ice and cell isolation was carried out within 1 hour of obtaining tissue. Primary EVTs were isolated from these samples based on an EVT-outgrowth based protocol established by Graham et al. and well-documented by other investigators (44–47). Briefly, villous tissue was finely minced and cultured at 37°C in RPMI 1640 medium containing 20% charcoal-stripped fetal bovine serum (FBS). After villous fragment attachment, EVT outgrowth occurred, and cells were separated from tissue during washing and passaging of the cells. Isolated EVTs were maintained in RPMI 1640 medium containing 20% FBS and 1% penicillin (100 U/mL)/streptomycin (100 U/mL) solution. EVT identity was confirmed by immunostaining for cytokeratin-7 and HLA-G. Cells were used within the first 3 passages.

#### Endothelial cells

A commercially available human primary endometrial microvascular endothelial cell line (HEMEC, ScienCell, #7010) cultured in recommended endothelial cell medium (ECM, ScienCell, #1001) was used between P1-5.

### IOC device preparation and cell seeding

Implantation-on-a-chip devices were fabricated as previously reported (42). Briefly, polydimethylsiloxane (PDMS) base polymer was mixed with the curing agent at a 10:1 weight ratio and then poured into a master mold with protruding features. It was then cured at for 2 h at 60°C. The resulting PDMS device was detached from the mold and underwent oxygen plasma treatment for 3 min and was attached to a PDMS-coated well plate. It was then sterilized under UV for 30 min and stored in an incubator until use. To stably maintain the mock ECM hydrogel in the middle lane, dopamine hydrochloride solution (2 mg/mL in pH 8.5 Tris-HCl buffer) was injected into the device, followed by incubation at 37°C for 2 h to coat the channel surface. After washing twice with water and drying completely, the mock ECM was injected into the middle lane for the experiment. To make the ECM hydrogel precursor solution, rat tail collagen Type-1 (8 mg/mL, Advanced Biomatrix) solution was prepared by mixing 10x PBS, 1N NaOH, and distilled water to achieve physiological pH, and then Matrigel (10 mg/mL, Corning, USA) was mixed with Col-1 solution at a ratio of 1:1 (v/v). ECM precursor solution was injected into the middle lane of the device and was incubated 37°C for 30 min to form an ECM hydrogel. After gelation, simultaneously with the injection of fibronectin solution (10 μg/mL in PBS) into the vascular channel, the device was tilted to allow seeding of EVTs (8 million cells/mL), facilitating their settlement and adhesion on the surface of the ECM hydrogel. After incubating for 30 min at 37°C, the fibronectin solution was removed from the vascular channel. Subsequently, Maternal ECs (10 million/mL) were seeded into the channel and further incubated for an additional 2 h at 37°C. Culture media were then supplied to the media reservoirs in the device for prolonged culture. Media were changed daily.

EVTs used in this study were fluorescently labelled by pre-incubating with 20 μM. CellTracker Green CMFDA (Thermo Fisher Scientific, USA) in RPMI supplemented 20% (v/v) FBS for 15 min at 37°C, respectively. To produce uNK cell or macrophage-containing devices, sorted uNK cells or macrophages were suspended in an ECM precursor solution at a density of 2 million cells/mL and injected into the middle lane of the device. Following incubation 30 min at 37°C, media reservoirs of the device were filled with 5% FBS supplemented RPMI and EGM-2MV medium. 400 ul of culture medium was added to the medium reservoirs connected to each chamber.

### Quantification of EVT invasion

Fluorescence images of EVTs were obtained from the ECM matrix region between the maternal vascular and fetal compartments of the IOC device. High magnification images were collected from three separate devices per each experimental group by using a confocal microscope (LSM 800, Carl Zeiss). EVT invasion was quantified by (i) the number of invading EVTs, (ii) the depth of EVT invasion, and (iii) the area of EVT invasion. To evaluate the cell number, EVTs in the ECM compartment were manually counted, and the average of total cell counts was plotted. The depth of invasion was determined by averaging the vertical distance that EVTs had travelled in the ECM scaffold. Analysis of invasion area was achieved by using the Analyze pixels function of ImageJ (NIH) with appropriate thresholding to measure the area of ECM hydrogel covered by invading EVTs.

### Statistical analysis

For IOC device experiments, one way ANOVA followed by multiple comparison testing was performed using Prism when combining all biological replicates and within each biological replicate, with a minimum of three independent devices for each experimental group (n=3).

## Results

### Identification of mid-secretory endometrium and first trimester decidual immune subclusters at high resolution

**Figure 1:**
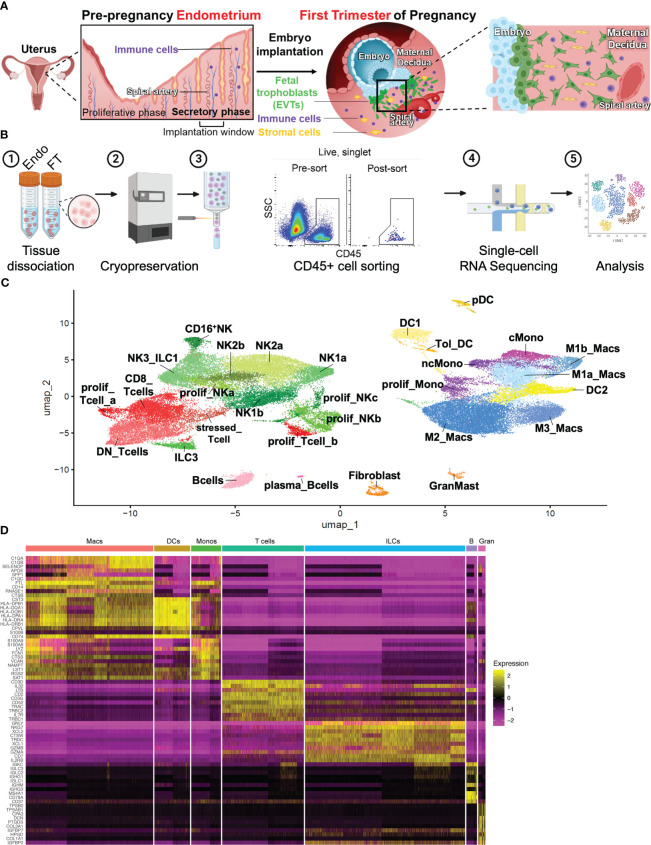
Combined immune landscape of the window of implantation endometrium and first-trimester decidua at high resolution. **(A)** Graphical representation of cellular changes that occur during the menstrual cycle and early pregnancy, including recruitment of immune cells into the uterine lining. **(B)** Experimental workflow. Four endometrial biopsies and four first trimester deciduae were enzymatically digested into single cell suspensions and cryopreserved. Frozen cells were then thawed, stained, and FACS-purified as CD45+ cells prior to scRNAseq and downstream analyses. Shown are raw flow cytometry data of pre- and post-sort purity of CD45+ cells. Events shown have undergone forward and side scatter gating, singlet discrimination, and live/dead discrimination. **(C)** 30 total clusters of CD45+ cells were identified. Subclusters are shaded according to supercluster: red, T cells; green, ILCs; pink, B cells; yellow, DCs; purple, monocytes; blue, macropahges. “Prolif” denotes proliferating; “DN”, double-negative (CD4-CD8-); “ILC”, innate lymphoid cell; “NK”, natural killer cell; “DC”, dendritic cell; “Tol_DC”, tolerogenic DC; “pDC”, plasmacytoid DC; “cMono”, classical monocyte”; “ncMono”, nonclassical monocyte; “Macs”, macrophages. **(D)** Heatmap representation of supercluster-defining is shown.

A major objective of this study was to comprehensively characterize the immune cell composition prior to and during very early pregnancy (**Figure 1A**). To do this, we obtained endometrial biopsies from four women between the ages of 23-33, with regular cycles and no known uterine pathology (**Supplementary Figure 1A**). By obtaining biopsies timed to coincide with the window of implantation, during the mid-secretory phase of the menstrual cycle, we examined the immune cells poised to participate in the very earliest stages of pregnancy. We compared these samples to first trimester pregnancy samples collected from four women between the ages of 29 and 32, undergoing elective termination between 5.8 weeks and 9.4 weeks gestation. No statistically significant differences between age and BMI were found between patients (**Supplementary Figure 1A**). Mid-secretory endometrial (MSE/Endo) and first-trimester decidual (FT) tissue was dissociated and frozen. Immediately before sorting, samples were thawed, and CD45^+^ uterine immune cell populations were sorted and processed for scRNA-seq (**Figure 1B**).

All samples were integrated into a single object, and single-cell expression profiles were generated using a cluster resolution of 0.9 (**Figure 1C**). Integration via *Seurat v4* mandates that any cluster found in one sample be present in each other sample, accounting for biologic variability between subjects and variation of gestational ages of the FT samples (48). Ensuring an appropriate number of cell clusters, or groups of cells that show transcriptomic similarity during scRNA-seq analysis is often subjective and determines accurate and reliable characterization of cell types. We began our analysis using a cluster resolution of 0.3 (not shown), which resulted in cell clusters of similar number and characteristics as those seen Vento-Tormo et al. (32), a seminal publication that first characterized the human maternal-fetal interface using scRNAseq. Since that work examined all cells present in the decidua, we increased our cluster resolution, taking into consideration our focus on only immune cell populations. We identified 30 distinct cell clusters, which could be grouped into the following superclusters based on expression of classic lineage genes: T cells (5 sub-clusters), innate lymphoid cells (ILCs, 10 sub-clusters), B cells (2 sub-clusters), Macrophages (4 sub-clusters), Monocytes (3 sub-clusters), Dendritic Cells (DCs, 4 sub-clusters), and Granulocytes/Fibroblasts (2 sub-clusters) (**Figures 1C, D**).

We found that the 4 MSE samples had modest variability among proportions of immune superclusters and subclusters (**Supplementary Figures 1B, C**). Prior scRNAseq data from FT deciduae demonstrated variability in cells recovered from individual donors (32). As expected, the 4 FT samples obtained from different gestational ages showed more variability, with macrophage, ILC, DC and T cell superclusters showing the greatest differences. We acknowledge that the greatest differences were between a sample isolated at 5 weeks, 6 days gestation (rich in macrophages, poorer in ILCs) from a White individual and a sample isolated at 11 weeks, 6 days gestation from a Black individual (poorer in macrophages, richer in ILCs).

### Identification of ILC subpopulations

#### Natural killer cells

Due to their abundance and importance in the preimplantation uterus and early first trimester decidua (15, 41), NK cells have been the focus of recent reports using scRNAseq (32, 49) or high-dimensional cytometry (33) to identify specific subsets present prior to and early in pregnancy. Multiple subsets have been found, and multiple naming schemes have been used in the literature. We thus aimed to integrate our current dataset with prior literature, with all immune subpopulations identified below also summarized in **Table 1**.

**Table 1:** Correlation of subclusters of pre- and post-implantation immune cells with previously described immune subtypes in the literature.

Supercluster	Subcluster	Closest existing subset(s)	Known or presumed protein markers based on enriched genes	Additional enriched genes
B cells	B cells	Mature inexperienced liver B cells (50)	CD19+	CD79A/B, CD22
B cells	Plasma B cells	Plasmablasts/plasmacells(50)		CD79A, MS4A1, JCHAIN, XBP1, TNFRSF17
DCs	DC1	Decidual DC1, CLEC9A+ DCs (51)	CLEC9A+HL^hi^	C1ORF54, XCR1, CADM1, CAMK2D
DCs	DC2	Decidual DC2, Non-inflammatory CD1c+ DCs (51)	CD1c+HLA^hi^	FCER1A,CLEC10A
DCs	pDCs	Plasmacytoid DCs (52)	BDCA2+IL3RA+	IRF7,IRF8,TCF4
DCs	ToIDCs	Tolerogenic DCs (53, 54)	CD80+PD-L1+IDO1^hi^	CCL19, CCL22
Fibroblasts/Granulocytes	Fibroblasts	Fibroblasts (55)	DCN+	SPARC, APOD, CALD1
Fibroblasts/Granulocytes	Mast cells	Mast cells (56)	CD117+ST2+	TPSAB1, CPA3
ILCs	CD16+NK	Decidual CD16^bright^ NK cells, Decidual NK4 cells (32, 57)	CD56^dim^ CD16^bright^	FGFBP2, SPON2, MYOM2
ILCs	ILC3	Decidual ILC3, LTi-like cells (58, 59)	CD117+CD127+CD56^+/-^NKp44^+/-^CD94-	IL7R, KIT, LTB, AHR, TOX2, TCF7, RORA, ID2
ILCs	NK1a	Decidual NK1 cells, adaptive-like CD56^bright^ NK cells (32, 33, 60)	CD56^bright^CD16^dim^ Eomes^hi^T-bet^hi^ CD49a^hi^CD39+	CSF1, SPINK2, ID3, PRF1, GZMB
ILCs	NK1b			TTN, CDHR1
ILCs	NK2a	Decidual NK2 cells (32, 33)	CD56^bright^CD16^dim^ CD39-CD103-KIR^low^ NKG2A/C/E+XCL1/2+	CCL5, CD9, ZNf683
ILCs	NK2b			CCL4, KLRB1, CD7, ZNF683
ILCs	NK3	Decidual NK3 cells, ILC1 (32, 33, 41, 49)	CD56^bright^CD16^dim^ CD103+CXCR4+CD161+NKp44+ Eomes^int^ T-bet^hi^	CCL3, CCL4, CCL5
ILCs	Proliferating NK-a	(Proliferative) decidual NK2/3 (32, 33)	CD56^dim^CD16^bright^Ki67+	Predominantly cell cycle genes
ILCs	Proliferating NK-b	(Proliferative) decidual NK1/2/3 cells (32, 33)		
ILCs	Proliferating NK-c	(Proliferative) decidual NK1/2/3 cells (32, 33)		
Macrophages	M1a	Decidual M1 macrophages, angiogenic TAMs (32, 40, 61)	CD14+CD11c^hi^	FCN1, OLR1, CLEC5A, VCAN, TIMP1, CTSB, CEBPB, EREG, VEGFA
Macrophages	M1b	Decidual M1 macrophages, inflammatory TAMs (32, 40, 61)	CD14+CD11c^hi^HLA-DR^lo^	IL1A/B, NLRP3, CASP1, IL1RN, PYCARD
Macrophages	M2	Decidual M2 macrophages, lipid-associated TAMs, regulatory TAMs (32, 40, 61)	CD14+CD11c^lo^HLA-DR^hi^	C1QA, C1QB, C1QC, APOC1, APOE, RNASE1, FOLR2, F13A1, TREM2, MRC1, CD163, MERTK HLA-DR/DQ, CD86, CX3CR1
Macrophages	M3	Decidual M3 macrophages (32, 61)	CD14+HLA-DR^hi^	APOC1, APOE, C1QA, C1QB, C1QC, TREM2, FOLR2, GPNMB
Monocytes	cMono	Decidual monocytes, classical monocytes (32, 62)	CD14+CD16-	VCAN, S100A8, S100A9, S100A12, CSF3R, LYZ, FOS, AREG, IL1B
Monocytes	ncMono	Decidual monocytes, non-classical monocytes (32, 62)	CD14-CD16+	FCGR3A, IFITM2, IFITM3, LST1, S100A4, S100A6, SERPINA1
Monocytes	proliferating Mono/macs	Proliferating decidual monocytes/macropahges (63)	CD14+Ki67+	Predominantly cell cycle genes
T cells	Activated/Stressed T cell	Highly activated CD8+ T cells (64)	CD3+CD8+	MALAT1, SYNE1, SYNE2, MTRNR2L8/12, AAK1, NKTR
T cells	CD8 T cells	Decidual CD8 T cells, CD8 T_CM_/T_EM_ (41, 65)		CD27, BCL11B, GZMA/K/M, TIGIT, TRGC2
T cells	DN T cells	Decidual DN T cells (41, 66)	CD3+CD4-CD8-	CD27, BCL11B, GZMA/K/M, TIGIT, IL7R, CD40LG, KLRB1
T cells	Proliferating T cell A	(Proliferating) decidual CD8 T cells	CD3+CD8+	Predominantly cell cycle genes
T cells	Proliferating T cell B	(Proliferating) decidual CD8 T cells		

Data are organized by supercluster. Shown are our proposed names for each subcluster and the closest existing subset(s) in the literature, along with transcriptional and phenotypic information for each subset.

**Figure 2:**
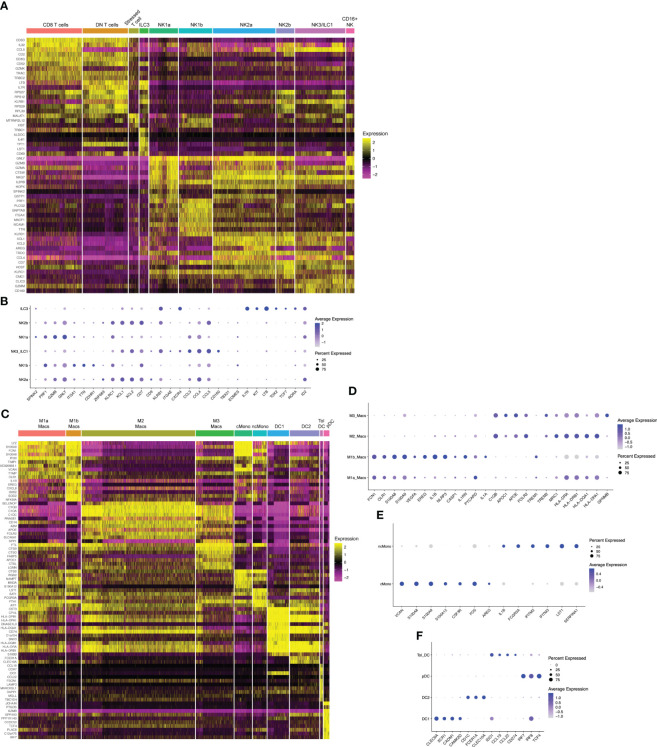
Hallmark genes of immune subclusters in the combined window of implantation endometrium and first-trimester decidua. Heatmap visualizations of cluster-defining genes across subclusters of **(A)** lymphocytes and **(C)** myeloid cells. Additional dot plot visualizations **(B, D–F)** of selected genes and subclusters are shown. Size of the dots indicates percent of total cells in each subcluster that express the indicated genes. Color of the dots correlates to the average level of expression of the indicated genes. B cells, mast cells, and fibroblasts are not shown due to these being rare cells in our dataset.

Previous scRNAseq data show a single cluster of NK1 cells, defined by cytotoxic genes PRF1, GZMB, GZMA, and GNLY, as well as KIRs and SPINK2 (32). By flow and mass cytometry, NK1 cells were defined by high expression of Eomes, T-bet, CD49a, and CD39 (32, 33). By mass cytometry, additional NK1 subsets could be found heterogeneously expressing KIRs (33). With the resolution of our current dataset, we find two clusters of transcriptionally-distinct NK1 cells that we denote NK1a and NK1b (**Figures 1C**, **2A, B**). Both express high levels of EOMES, PRF1, and GZMB (**Figures 2A, B**). In addition, NK1a and NK1b cells are rich in genes encoding integrins ITGA1 (CD49a), ITGAD (CD11d), and ITGAX (CD11c), as well as AFAP1L2 (encoding the adaptor protein XB130), a gene expression profile resembling adaptive-like CD56^bright^ NK cells (60). The NK1a cluster is particularly enriched for cytolytic genes, CSF1 (M-CSF), SPINK2, and ID3, while NK1b cells are specifically enriched for TTN (encoding the structural protein titin) and CDHR1 (encoding a cadeherin-related adhesion molecule).

NK1 and NK2 cells compose the majority of uNK cells in first trimester decidua, both increasing in frequency from secretory phase endometrium (49). We next examined NK2 subsets. Previous scRNAseq and flow/mass cytometry showed a single decidual NK2 subset, defined as CD39-CD103- cells expressing high levels of NKG2A, XCL1, and XCL2 but low levels of KIRs (32, 33). We found two NK2 subsets by scRNAseq (**Figures 1C**, **2A, B**). Both NK2 subsets, denoted NK2a and NK2b cells, exhibited high levels of ZNF683 transcript (**Figure 2B**), encoding HOBIT, previously implicated in ILC1 development and function in mice (67) and conventional NK cell development in humans (68). We find NK2a and NK2b cells further defined by enrichment of KLRC1 (NKG2A), KLRC2 (NKG2C), KLRC3 (NKG2E), as well as XCL1 and XCL2. With the exception of KIR2DL4, NK2a and NK2b cells exhibited low expression of KIRs. While NK2a cells expressed higher CCL5 (RANTES), NK2b cells expressed higher CCL4 (MIP-1β). NK2a cells also expressed more cytolytic transcripts, particularly GZMB and PRF. NK2a cells expressed more CD9, while NK2b cells were defined by higher expression of KLRB1 (encoding CD161) and CD7.

The NK3 subset, defined phenotypically as CD103^+^CD56^bright^ cells, appear to peak during the secretory phase and decline as a proportion of total NK cells during the first trimester (32, 41, 49). Our previous work demonstrates that NK3 cells are numerically and functionally impaired in the setting of a high-risk preimplantation endometrium (41). Previous scRNAseq and flow cytometry data showed NK3 cells as high expressers of the integrin CD103 (ITGAE), as well as CXCR4 and CCL5 (32, 33). Due to expression of CD103, high expression of T-bet, intermediate expression of Eomes, and high expression of NKG2D, CD161, and NKp44, NK3 cells were shown to resemble intraepithelial ILC1s by mass cytometry (33). In prior datasets, NK3 cells appear to produce a number of chemokines in the steady state and upon stimulation, namely CCL3 (MIP-1α), CCL4 (MIP-1β), and CCL5 (RANTES) (32, 33). In our current scRNAseq dataset, we also find one cluster of NK3/ILC1 cells,enriched for CD160, KLRB1 (CD161), CCL3, CCL4, and CCL5 (**Figures 2A, B**).

The dominant population of uterine NK cells exhibits a CD56^bright^CD16^dim^ surface phenotype, while the dominant population of peripheral blood NK cells are CD56^dim^CD16^bright^. A relatively small population of CD16^bright^ NK cells have been found in the decidua by scRNAseq and by flow cytometry (32, 57). Upon stimulation with IL-12, IL-15, and IL-18, the majority of cultured decidual CD16^bright^ NK cells produced IFNγ, whereas only a minority of cultured decidual CD56^bright^ NK cells produced IFNγ (57). The proportion of decidual CD16^bright^ NK cells were modestly decreased in the term decidua in the setting of preeclampsia and increased in number in the peripheral blood and decidua of recurrent pregnancy loss patients (69). We also find a population of CD16+NK cells (**Figures 1C**, **2A**) defined by high expression of FCGR3A (CD16), FGFBP2, SPON2, and MYOM2.

Proliferative NK precursor populations have been identified in the decidua by scRNAseq and by Ki67 detection with mass cytometry (32, 33). We now show three clusters of NK cells predicted to be proliferating in S phase or G2/M phase (**Figure 1C**, **Supplementary Figures 2A, B**). The cluster we denote Proliferating NK-a (prolif_NKa) shares marker genes CD160, ITM2C, GZMM, KRT81, KRT86, IGFBP2, and TNFRSF18 with predominantly NK2 and NK3 subsets. In contrast, the subsets we denote prolif_NKb and prolif_NKc shared marker genes PRF1, GZMA, GZMB, GNLY, EOMES, KLRC1, KLRD1, KLRK1, XCL1, and XCL2 with all NK subsets, including NK1 cells. The proliferative NK cell subsets may represent replicating cells already committed to various NK lineages, or they may represent precursor populations that give rise to particular types of NK cells. Substantial amounts of dividing NK cells suggest that the preimplantation endometrium and early pregnant decidua support NK cell proliferation, likely with IL-15 and other NK cell mitogens (31).

Taken together, our data align well with prior datasets and extend understanding of the immune landscape of the preimplantation endometrium and first trimester decidua with our higher resolution and focused assessment of immune cells. Our data also support the notion that critically important NK cell populations are already in place prior to pregnancy in implantation-window endometrium and play a critical role in initiating and regulating early placentation (42).

#### Type 3 innate lymphoid cells

Both natural cytotoxicity receptor (NCR)+ and lymphoid tissue-inducer (LTi)-like decidual ILC3 cells have been identified by flow cytometry in previous reports (58, 59). These cells express RORγt protein and produce IL-17, IL-22, and GM-CSF upon stimulation *in vitro*. We find that ILC3s express high levels of *IL7R*, *KIT*, *LTB*, *AHR*, as well as *TOX2*, *TCF7*, *RORA*, and *ID2* (**Figures 2A, B**), all associated with ILC3s and LTi-like cells. In our data and others’ scRNAseq data from early pregnancy (32), decidual ILC3s express minimal *RORC* (encoding RORγt), *IL17* transcripts, *IL22*, and *CSF2* (encoding GM-CSF). These data reveal discrepancies between transcript- and protein-level data and suggest that ILC3/LTi-like cells in the uterine lining prior to and during early pregnancy do not produce traditional ILC3-associated cytokines in the steady state.

### Characterization of myeloid subpopulations

#### Macrophages

Consistent with prior data, we find that macrophages are highly abundant in implantation window endometrium and in the early decidua (**Figure 1C**). They are especially abundant in the decidual basalis, the site of implantation, equaling or exceeding the frequency of NK cells by the first trimester of pregnancy (63). We resolved 4 clusters of decidual macrophages by scRNAseq. Although macrophage polarization into M1 (“pro-inflammatory”) and M2 (“anti-inflammatory or tissue-repair”) subsets is commonly used as a conceptual framework, macrophages exhibit substantial context- and tissue-specific heterogeneity. In the interest of unifying nomenclature previously used to describe macrophages in pregnancy found by scRNAseq, we chose to use M1/M2 designations below, plus an M3 designation previously used to describe maternal macrophages that were found in close association with fetal villi (32).

We found 2 distinct clusters of macrophages, denoted M1a and M1b, that exhibit expression of canonical M1 genes (**Figures 1C**, **2C, D**). M1a and M1b macrophages expressed high levels of S100A8 and S100A9 (**Figures 2C, D**). M1a macrophages were also enriched for FCN1, OLR1, CLEC5A, VCAN, TIMP1, CTSB, CEBPB, EREG, and VEGFA, a gene expression profile resembling angiogenic tumor-associated macrophages (61). The M1b subset was highly enriched in proinflammatory genes and those associated with activation of the NLRP3 inflammasome, such as IL1B, NLRP3, CASP1, IL1RN, PYCARD, and IL1A (**Figures 2C, D**). M1b macrophages have overlapping features with inflammatory tumor-associated macrophages (61) and approximate the M1 subset of decidual macrophages expressing low levels of HLA-DR by imaging mass cytometry (40). Based on their proinflammatory gene expression profile, both M1a and M1b macrophage clusters align with CD11c^hi^ or CCR2^+^CD11c^hi^ subsets of macrophages that have been described by other groups (27, 28).

M2 macrophages in our dataset (**Figures 1C**, **2C, D**) aligns with the M2 macrophage subset in previous scRNAseq data of the first trimester decidua (32). M2 macrophages exhibit high levels of MRC1 transcript, encoding the canonical M2 marker CD206, and low levels of ITGAX, encoding CD11c (**Figures 2C, D**). These cells are likely contained within the CD11c^low^ decidual macrophage subset previously reported (27). More recent data showed that CD14+ macrophages expressing CD206 and CD163 expressed RNASE1 and FOLR2 (39), also marker genes within our M2 subset (**Figures 2C, D**). Based on enrichment of C1QA, C1QB, C1QC, APOC1, APOE, RNASE1, FOLR2, F13A1, TREM2, MRC1, CD163, MERTK, CTSB/D/L, LIPA, and ATF3, our M2 macrophages exhibit characteristics of lipid-associated TAMs. Based on expression of class II major histocompatibility complex (MHC-II) HLA-DR and HLA-DQ genes, CD86, CCL2, CX3CR1, LGALS9, and ITGA4, our M2 macrophages also exhibit characteristics of regulatory TAMs (61).

The gene expression profile of M3 macrophages overlapped closely with M2 macrophages, with marker genes such as APOC1, APOE, C1QA, C1QB, C1QC, TREM2, and FOLR2, among others (**Figures 1C**, **2C, D**). It could be distinguished from M2 macrophages transcriptionally by specific enrichment of GPNMB (**Figure 2D**), encoding a transmembrane glycoprotein that can be cleaved at the cell membrane and bind to a variety of receptors to modulate immune activation (70).

Of note, we identified a subset of cells that clustered with macrophages but were called part of more “distant” NK cells on the UMAP by the clustering algorithm (**Figure 1C**). With the current resolution of this dataset, we were unable to separate this subcluster of macrophages for further analysis. We suspect these represent a population of uterine macrophages we previously described, which express CD122 (IL2RB), the β chain of the IL-2/IL-15 receptor complex, as well as cytolytic molecules (71). Another group recently confirmed our findings of decidual macrophages co-expressing NK cell markers using imaging mass cytometry (40).

Proliferating macrophage/monocyte-lineage cells were evident in our dataset (**Figure 1C**, **Supplementary Figures 2A, B**), consistent with data showing that CD14+ cells within the decidua basalis express Ki67 and incorporate EdU in explants of decidua basalis (63). In contrast, proliferating CD14+ cells were not evident in the decidua parietalis. These data lend support to the notion that macrophage-lineage cells proliferate *in situ* in the decidua prior to and during early pregnancy. These data are also in line with mouse studies that show resident macrophages undergo proliferation *in situ* in the myometrium in an M-CSF-dependent fashion, while macrophage proliferation *in situ* in the decidua proceeds at a lower level and in an M-CSF-independent manner (72).

#### Monocytes

In mice, circulating monocytes traffic to the decidua during early pregnancy, extravasate into the tissue, and adopt a tissue macrophage phenotype (71, 72). We find 2 clusters of monocytes in the implantation window endometrium and the first trimester decidua (**Figures 1C**, **2C**). Classical monocytes closely resembled classical CD14^+^CD16^-^ monocytes in the blood (62), as they were marked by high levels of VCAN, S100A8, S100A9, S100A12, CSF3R (G-CSFR), LYZ, FOS, AREG, and IL1B (**Figure 2E**). Similarly, non-classical decidual monocytes resembled their blood CD14^-^CD16^+^ counterparts (62), enriched for FCGR3A (CD16), IFITM2, IFITM3, LST1, S100A4, S100A6, and SERPINA1.

#### Dendritic cells

We resolved 4 clusters of decidual dendritic cells (DCs) (**Figures 1C**, **2C**). Similar to CLEC9A+ human DCs in the blood of healthy donors (51), our DC1 cluster is enriched for CLEC9A, C1ORF54, XCR1, CADM1, and CAMK2D, as well as MHC-II genes (**Figure 2F**). Our DC2 cluster is marked by very high levels of MHC-II genes, CD1C, FCER1A, and CLEC10A. Based on this gene expression profile, these DCs are most similar to non-inflammatory CD1C^+^ DCs in the blood (51). We denote a third cluster tolerogenic DCs, expressing high levels of IDO1, CCL22, CCL19, CD80, and CD274 (PD-L1). These cells phenocopy tolerogenic DCs described in other contexts, particularly lymph node-resident tolerogenic DCs that are induced by T regulatory cells and upregulate IDO in response to autologous apoptotic cells or CCL19 (53, 54). Finally, we find a fourth cluster of cells resembling blood plasmacytoid DCs, based on expression of IL3RA, IRF7, and CLEC4C, as well as master pDC transcription factors IRF8 and TCF4 (52).

### Characterization of adaptive lymphocyte subpopulations

#### T cells

While T cells are thought to mediate tolerance of the allogeneic fetus later in gestation, roles for T cells during the implantation window and early pregnancy are underexplored. Our group previously showed that, in contrast to peripheral blood, the two dominant populations of CD45+CD3+CD56- T cells in implantation window endometrium by flow cytometry are either CD8+ or DN T cells (41). Our present scRNAseq data show that the two largest clusters of T cells are defined by CD8A and CD8B (CD8+ T cells) or by the absence of CD8A, CD8B, and CD4 (CD4-CD8-, or double-negative [DN] T cells) (**Figures 1C**, **2A**).

The CD8+ T cell cluster was further defined by high expression of CD27, the transcription factor BCL11B, select granzymes (GZMA, GZMK, and GZMM), and the inhibitory molecule TIGIT. In agreement with other recent scRNAseq datasets of cytotoxic lymphocytes (65), CD8+ T cells also expressed TRGC2 but not TRDC or TRGC1 (not shown). At term, DN T cells are also evident in the decidua basalis and parietalis (66). Of term DN T cells, nearly half are TCRγδ+ by flow cytometry. We now find in the preimplantation and first-trimester uterus, the DN T cell cluster was enriched for CD27, BCL11B, GZMA, GZMK, GZMM, and TIGIT, similar to the CD8+ T cell cluster. A minority of DN T cells expressed TRDC, TRGC1, or TRGC2, while a majority of DN T cells expressed TRAC, TRBC1, and TRBC2, suggesting this cluster contained a large proportion of TCRαβ+ T cells (**Figure 2A**). However, extremely few cells within the DN T cell cluster expressed CD8A or CD8B. Instead, scattered cells within the DN T cell cluster expressed CD4 transcript, suggesting that uterine DN T cells may repress surface expression of CD4. Further distinguishing them from CD8+ T cells, DN T cells expressed high levels of IL7R, CD40LG, and KLRB1 transcripts.

A third cluster of T cells we denoted activated/stressed T cells expressed TRAC and TRBC, suggesting these are TCRαβ+ T cells (**Figure 2A**). They express the lncRNA MALAT1, as well as SYNE1, SYNE2, and MTRNR2L12, which have been associated with highly activated CD8+ T cells found in the setting of COVID-19 (64). Also enriched in this cluster was the natural killer triggering receptor NKTR, associated with IL-2 activation, and AAK1, implicated in chemokine receptor expression and trafficking to the tumor microenvironment (73). However, this cluster exhibited lower expression of cytotoxicity markers and higher expression of MTRNRL8, recently found to be associated with metabolic stress in the setting of mitochondrial dysfunction (74).

Two clusters of proliferating T cells were found (**Figure 1C**, **Supplementary Figures 2A, B**). The first, denoted proliferating T cell A was clustered nearest to the CD8 and DN T cell populations (**Figure 1C**). This cluster is predicted to be in S phase and expresses high levels of marker genes associated with CD8 T cells (**Supplementary Figures 2A, B**). Another subpopulation, denoted proliferating T cell B, whose marker genes are characteristic of CD8 T cells were predicted to be in G2/M phase and clustered closer to proliferating NK cell subsets also predicted to be in G2/M phase. Taken together, these data show that the preimplantation endometrium and first-trimester decidua contain unique subpopulations of T cells.

#### B cells

We find two distinct clusters of B cells (**Figure 1C**), which have been found to play increasingly complex roles in pregnancy and fetomaternal tolerance (75, 76). The first cluster we find is enriched for pan-B cell genes CD79A, CD79B, and CD22, as well as several other genes that overlap with mature, inexperienced B cells found in the liver (50). The second cluster resemble plasma cells, highly enriched for Ig heavy and light chain genes, CD79A, MS4A1, JCHAIN, XBP1, and TNFRSF17, similar to plasma cells or differentiating plasmablasts in other datasets (50).

### Characterization of additional CD45+ subpopulations

#### Fibroblasts and granulocytes

With high expression of DCN, CALD1, APOD, and SPARC (**Figure 1C**), we denoted one small cluster as fibroblasts (55). Our data suggest the presence of a small cluster of mast cells, due to expression of hallmark genes (56) such as TPSAB1, CPA3, KIT, and IL1RL1. No granulocytes other than mast cells were were found in our dataset.

### Comparison of data quality and gene expression from fresh and cryopreserved endometrial immune cells

For this study, human samples from secretory phase endometrium and first trimester decidua were obtained when patient samples became available, presenting logistical challenges if fresh tissue samples were required for each downstream assay, including scRNA sequencing. In particular, a bank of viable, cryopreserved cells offers the opportunity to concurrently perform transcriptional and functional studies. To assess the effect of cryopreservation on differences in immune cell composition and gene expression in dissociated uterine tissue, cells were isolated from one endometrial biopsy obtained during the implantation window (**Supplementary Figure 3A**). Half of the dissociated cells were immediately processed and sorted for viable CD45+ cells using fluorescence-activated cell sorting (FACS). After FACS, sorted immune cells from freshly dissociated cells were processed up to the encapsulation stage after reverse transcription and stored at -20°C. The remaining cells were cryopreserved as described in the Methods section. After 1 week of cryopreservation, the frozen single-cell suspension was thawed, sort-purified for viable CD45+ cells and similarly processed for encapsulation. cDNA amplification, library preparation and sequencing for both fresh and frozen samples were then carried out concurrently. Both fresh and frozen samples showed high viability and quality control analysis indicated similar levels of genes per cell, UMIs per cell and mitochondrial genes per cell.

We first compared cell composition by carrying out cell cluster analysis in fresh and frozen/thawed samples. Given limitations in total cell number from one total endometrial biopsy, we classified cells into 5 superclusters representing major immune cell types – Myeloid (containing cells resembling monocytes, macrophages and dendritic cells), B cells, Granulocytes, T cells and ILCs (**Supplementary Figures 3B, C**). In this one endometrial sample, we find that the ILC cluster represented a lower proportion of total CD45+ cells in the frozen sample, with the myeloid cluster compensating with a higher proportion of total CD45+ cells in the frozen sample (**Supplementary Figure 3D**). The less abundant superclusters did not show any obvious differences in abundance between fresh and frozen/thawed processing.

We next asked if gene expression profiles were consistent between fresh and frozen/thawed samples. At the supercluster level, T cells, ILCs and myeloid cells exhibited between 100-300 DEGs between conditions, using a fold change cutoff of a 25% increase or decrease in expression (**Supplementary Figure 3E**). The top 20 DEGs in fresh and frozen/thawed myeloid cells and ILCs are provided in **Supplementary Figure 3F**. For instance, the frozen myeloid cluster was enriched in FOLR2 and APOE. These data are consistent with either an overrepresentation of M2/M3 cells (relative loss of M1 cells) in the frozen dataset or direct effects of freezing on detection of those transcripts.

Similarly, SPINK2 and GNLY were enriched in fresh ILCs, suggesting that NK1 cells may be underrepresented after freezing or that freezing has direct effects on those transcripts. Population- and gene-level changes between processing methods should be considered to enhance rigor and reproducibility of these data. However, combined with the above discussion closely aligning aspects of our data with prior literature, these data taken all together support that immune cells from uterine samples cryopreserved after cell dissociation are similar to freshly sorted and isolated cells.

### Population-level changes across decidual lymphoid and myeloid subpopulations after embryo implantation

**Figure 3:**
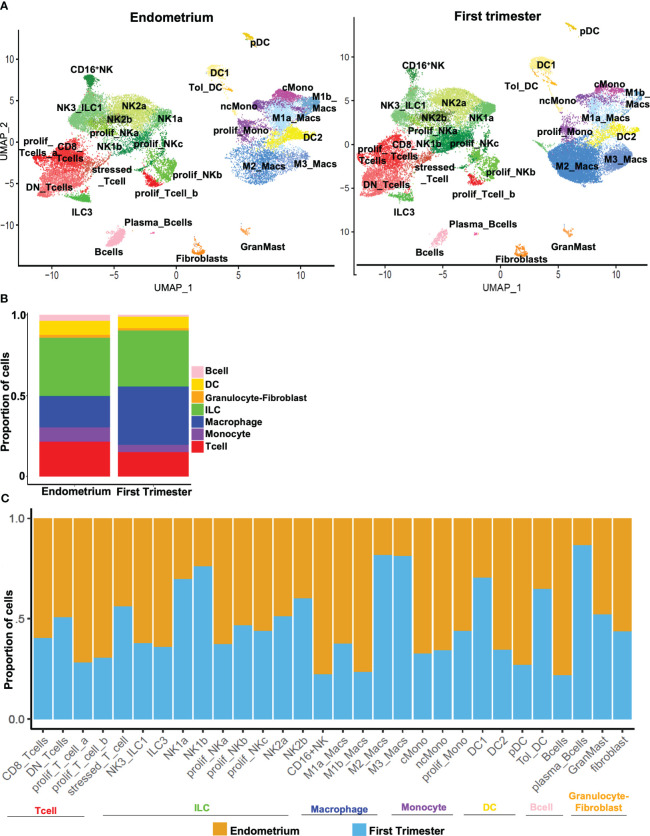
Dynamic changes in subpopulations of immune superclusters and subclusters in response to embryo implantation. **(A)** UMAP plots showing subclustering of CD45+ cells meeting quality metrics, recovered from mid-secretory endometrium (left) and first trimester decidua (right). ILC, innate lymphoid cells. **(B)** Stacked bar charts showing proportion of total CD45+ cells represented by indicated superclusters in pre-implantation endometrium and first trimester decidua. **(C)** Stacked bar charts showing proportion of total cells in indicated subcluster (x-axis) recovered from endometrium (orange bars) or first trimester decidua (blue bars).

After comprehensively identifying immune subsets present in the uterine lining during the window of implantation and during the first trimester of pregnancy (**Figure 3A**), we sought to compare the abundance of pre- and post-implantation immune cells. At the supercluster level, we observed a relative increase in the proportion of macrophages and a relative decrease in the proportion of monocytes post-implantation (**Figures 3A, B**). Only modest differences were evident in the overall proportions of pre- and post-implantation innate and adaptive lymphocytes.

We then identified trends in population density after implantation across all subclusters of immune cells. The decrease in the overall proportion of monocytes post-implantation was driven by decreases in abundance of both classical and nonclassical monocytes (**Figure 3C**). The increase in the overall proportion of macrophages evident in first-trimester tissue was driven by increases in both M2 and M3 macrophages that outweighed decreases in M1a and M1b macrophages. Compared to implantation-window endometrium, first trimester decidua contained modestly more DC1s and tolerogenic DCs and fewer DC2s and pDCs. Consistent with a recent report (49), we found proportions of NK2a and NK2b cells were similar pre- and post-implantation, proportions of NK1a and NK1b cells increased post-implantation, and the proportions of NK3/ILC1s and ILC3s both decreased in post-implantation deciduae. We further found that CD8+ T cells were modestly more common in pre-implantation endometrium, while proportions of DN T cells and stressed/activated T cells were unchanged in the pre- and post-implantation uterine lining. Proliferative subsets of both innate lymphocytes and T cells were recovered more frequently from implantation-window endometrium. B cells were more evident pre-implantation, while rarer plasma-like B cells were more evident post-implantation. We recovered similar proportions of fibroblasts and mast cells from implantation-window endometrium and first trimester decidua.

### Gene expression changes across decidual lymphoid and myeloid subpopulations after embryo implantation

**Figure 4:**
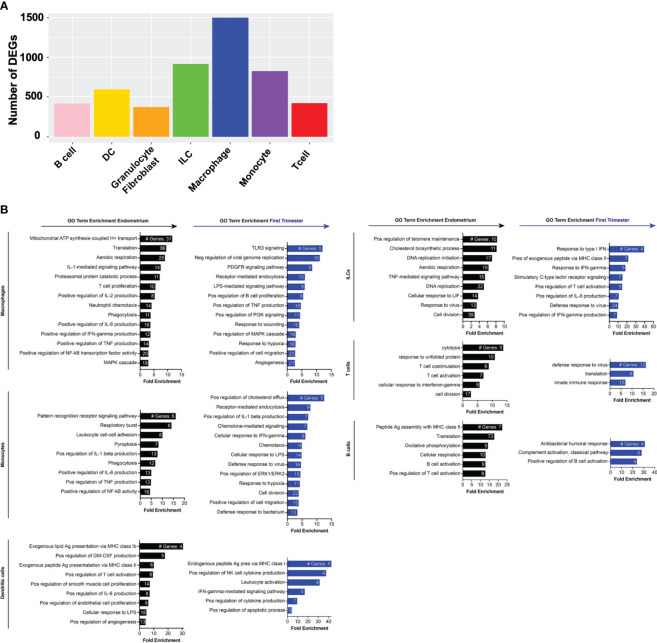
Gene expression changes across superclusters in response to embryo implantation. **(A)** Bar graph showing absolute number of significant differentially expressed genes (DEGs) between endometrial and first trimester decidual CD45+ cells in indicated superclusters. Significant genes are defined as having an adjusted p value of <0.05 and an absolute fold change of 25% or greater (either ≥25% enriched in endometrium or ≥25% in decidua). **(B)** Gene ontology (GO) analysis of DEGs enriched in indicated endometrial (black) or first trimester decidual (blue) superclusters. GO terms significantly enriched are shown (false discovery rate [FDR] value ≤0.05). The x-axis shows fold enrichment of the indicated GO term on the y-axis. Embedded in the bars are the number of DEGs contributing to each GO term. If applicable, GO terms were shortened due to space restrictions and/or combined into one term that contained the most enriched genes. For instance, “negative regulation of viral genome replication”, “response to virus”, “viral entry into host cell”, and “defense response to virus” are GO terms that contain highly overlapping genes. Thus, only one of the terms was included. Additionally, such broad GO terms as “immune system process” and “inflammatory response” were not included in a dataset consisting only of immune cells.

Key cell types involved in implantation may change in number, gene expression profile, or both. In addition to exhibiting numerous changes in population density, macrophages also accounted for the most DEGs among the superclusters in our dataset, followed by ILCs, monocytes, and T and B lymphocytes (**Figure 4A**). To uncover gene signatures associated with the window of implantation MSE samples or the first trimester, we performed gene ontology analysis using the Database for Annotation, Visualization and Integrated Discovery (DAVID) (77, 78) on genes enriched in endometrial or first trimester decidual immune superclusters, respectively. Across all MSE myeloid and lymphoid superclusters, we observed enrichment of genes associated with aerobic respiration, proliferation, and cell division (**Figure 4B**). MSE mononuclear phagocytes were broadly enriched for genes associated with NF-κB activity, production of the classical proinflammatory cytokines IL-1, IL-6, and TNF, and activation of lymphocytes. Pre-implantation ILCs were enriched for genes associated with TNF and LIF signaling. Endometrial ILCs were also enriched in genes associated with cholesterol metabolism, in agreement with a previous investigation of bulk endometrial and decidual NK cell transcriptomes by microarray (29). T cells in the pre-implantation endometrium exhibited high levels of cytolytic genes, as well as those associated with T cell costimulation and activation.

In contrast to pre-implantation immune superclusters, multiple post-implantation decidual superclusters were found enriched in interferon (IFN) stimulated genes (ISGs) (**Figure 4B**). In addition to ISGs, macrophages and monocytes also expressed families of genes in PI3K and MAPK activation pathways, hypoxia response pathways, proangiogenic pathways and classical proinflammatory pathways. Decidual dendritic cells upregulated genes associated with Class I MHC antigen presentation and activation of NK cells and other leukocytes. ILCs, T cells, and B cells also expressed ISGs, as well as collections of genes associated with innate and adaptive immune activation.

### Specific gene expression changes across pre- and post-implantation macrophage and NK subsets

**Figure 5:**
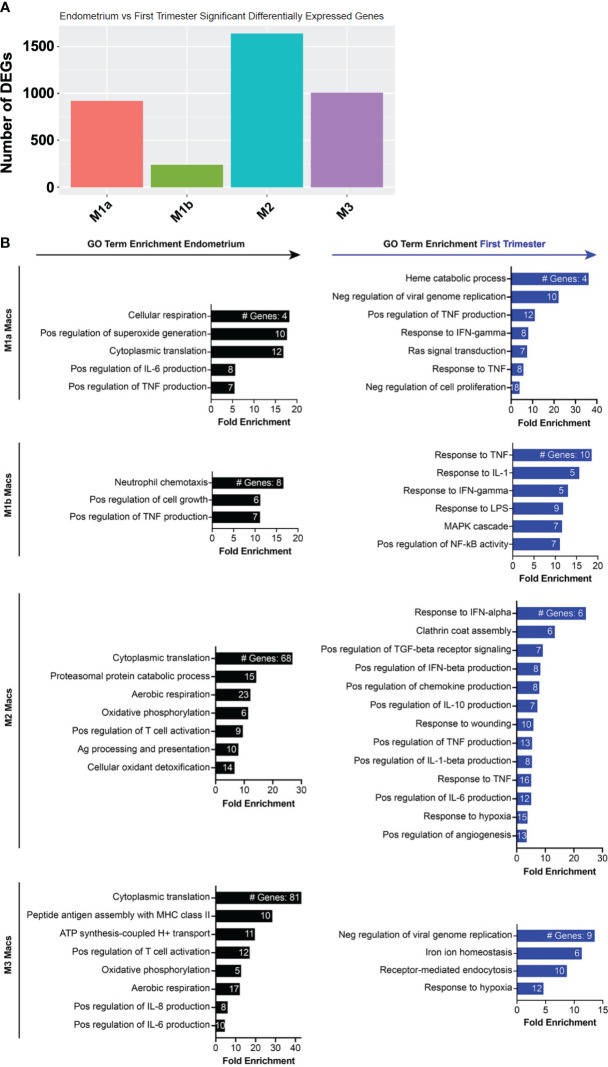
Inflammatory and metabolic pathways are enriched in pre-implantation endometrial macrophages, while interferon-stimulated, hypoxia, and inflammatory response genes are enriched in first trimester decidual macrophages. **(A)** Bar graph showing absolute number of significant differentially expressed genes (DEGs) between indicated endometrial and first trimester decidual macrophage subclusters. Significant genes are defined as having an adjusted p value of <0.05 and an absolute fold change of 25% or greater (either ≥25% enriched in fresh or ≥25% in frozen). **(B)** Gene ontology (GO) analysis of DEGs enriched in indicated endometrial (black) or first trimester decidual (blue) macrophage superclusters. Detailed information describing the GO term enrichment bar graphs is provided in the caption to **Figure 4B**.

Although NK cells and macrophages are the two most abundant cell types in the uterine lining during the implantation window, how they change transcriptionally after arrival of the embryo is not known. We thus compared the pre- and post-implantation transcriptional profiles of each of the four macrophage subsets we identified. M2 macrophages contained the most DEGs of the four subsets, and M1b macrophages contained the fewest (**Figure 5A**). The top 20 genes induced in M1a macrophages post-implantation were dominated by ISGs (**Figure 5B**, **Supplementary Figure 4**). Additional genes upregulated in this subset are involved in heme metabolism and the response to hypoxia. Post-implantation M1b macrophages upregulated canonical proinflammatory response genes, such as NLRP3, TNF, IL-18, and numerous chemokines downstream of NF-κB signaling. Decidual M2 macrophages also induced multiple ISGs, as well as numerous genes involved in production of IL-10, response to hypoxia, and regulation of angiogenesis. Similar to M2 macrophages, decidual M3 macrophages induced many ISGs and hypoxia response genes.

**Figure 6:**
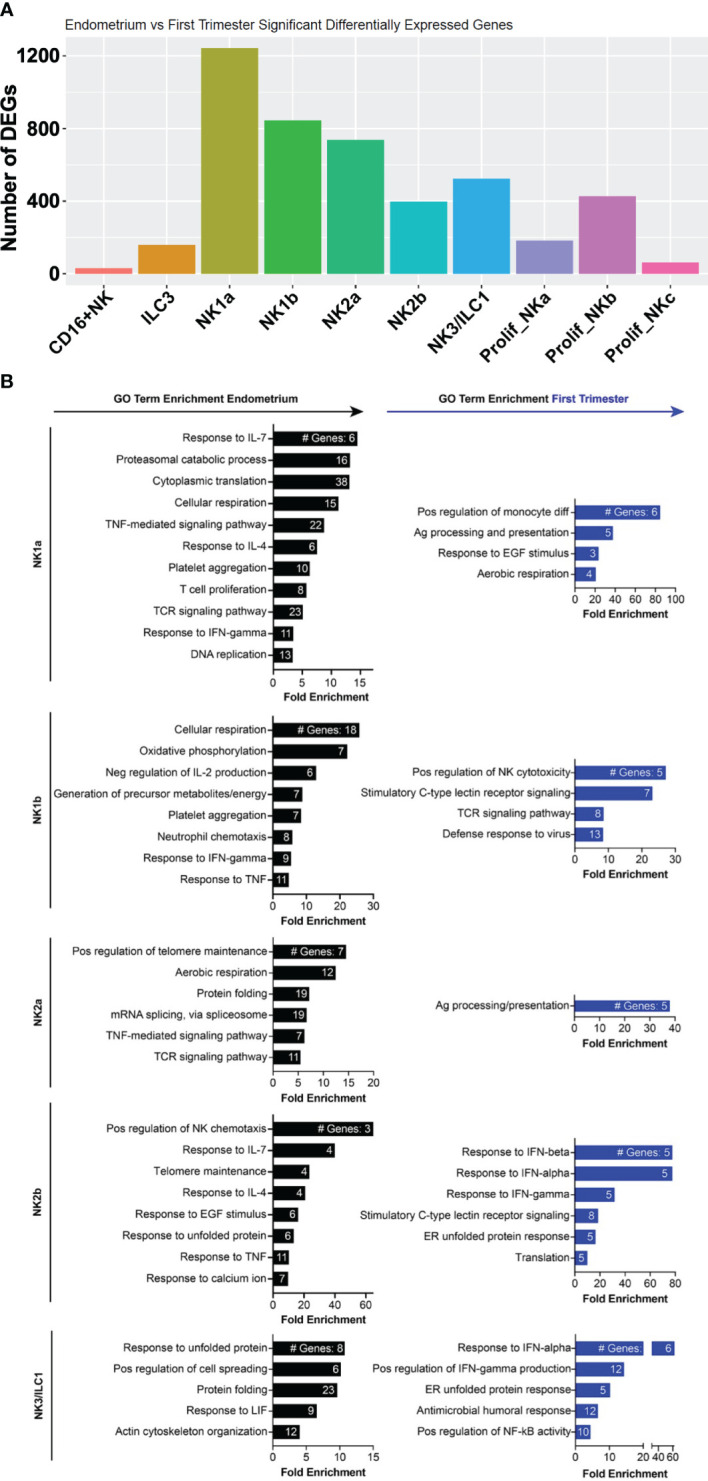
Metabolic, chemotactic, and inflammatory response pathways are enriched in pre-implantation endometrial NK cells, while interferon-stimulated genes are enriched in first trimester decidual ILC subsets. **(A)** Bar graph showing absolute number of significant differentially expressed genes (DEGs) between indicated endometrial and first trimester decidual ILC subclusters. Significant genes are defined as having an adjusted p value of <0.05 and an absolute fold change of 25% or greater (either ≥25% enriched in fresh or ≥25% in frozen). **(B)** Gene ontology (GO) analysis of DEGs enriched in indicated endometrial (black) or first trimester decidual (blue) ILC superclusters. Detailed information describing the GO term enrichment bar graphs is provided in the caption to **Figure 4B**.

Among ILCs, the NK subsets exhibited the most DEGs from pre- to post-implantation (**Figure 6A**). Decidual NK1b cells induced numerous killer lectin-like receptor genes and ISGs (**Figure 6B**, **Supplementary Figure 5**). Although pre- and post-implantation NK2a cells differentially expressed over 700 genes, they were scattered over multiple different pathways, resulting in only one gene ontology term, antigen processing and presentation, significantly enriched in this subset (**Figures 6A, B**). The top gene upregulated post-implantation in NK2a cells was AREG, encoding amphiregulin (**Supplementary Figure 5**). Like NK1b cells, decidual NK2b cells were enriched in ISGs and killer lectin-like receptor genes (**Figure 6B**, **Supplementary Figure 5**). NK3/ILC1 cells also strongly induced ISGs post-implantation, as well as genes involved in positive regulation of NF-κB and in the antimicrobial response, including IL-32. Data from NK1a cells may be biased by a population of cells that appeared post-implantation and clustered with macrophages (**Figure 3A**), so we interpret the NK1a DEG data with that caveat. Among the most upregulated genes in NK1a cells in the decidua were the cluster-defining gene SPINK2 and AREG (**Supplementary Figure 5**).

### Pre- and post-implantation macrophages and monocytes promote EVT invasion

Placentation requires invasion of a specialized trophoblast subset, the extravillous trophoblasts (EVTs), into the uterus towards maternal spiral arteries. EVTs transform spiral arteries into low-resistance, high-capacitance vessels, which nourish the fetus throughout gestation (79–82). To explore how endometrial and decidual macrophages and NK cells interact with trophoblasts to promote placentation, we developed and validated an implantation-on-a-chip device (IOC) to investigate roles for human uterine immune cells in EVT invasion ex vivo (41, 42). On the IOC, cultured primary EVTs migrate through a mock extracellular matrix (ECM), toward maternal ECs. We previously showed that different cell types embedded within the matrix differentially affect EVT invasion. For instance, decidualized stromal cells inhibit invasion, while pre-pregnancy uNK cells isolated from MSE promote invasion (42).

**Figure 7:**
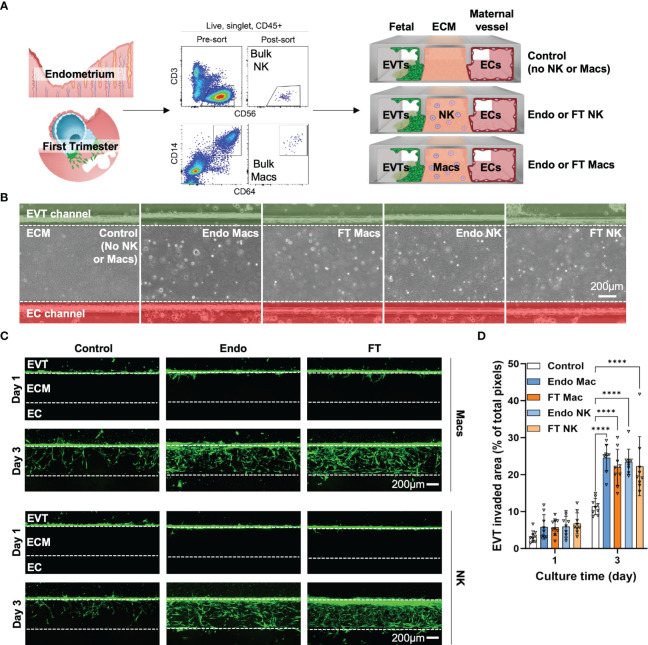
Pre- and post-implantation bulk CD14+CD64+ macrophages/monocytes promote invasion of primary EVTs as strongly as pre- and post-implantation bulk NK cells ex vivo on an implantation-on-a-chip device. **(A)** Graphical representation of the experimental approach taken to prepare and load purified endometrial and decidual bulk NK cells (CD56+CD3- cells, top flow cytometry plots) and bulk macrophages (CD14+CD64+, bottom flow cytometry plots) onto the implantation-on-a-chip (IOC). Shown are raw flow cytometry data of pre- and post-sort purity of NK cells and macrophages. Events shown have undergone forward and side scatter gating, singlet discrimination, live/dead discrimination, and CD45+ gating. “Fetal” denotes the channel of the IOC containing fluorescently labeled fetal EVTs, “ECM” denotes the channel of the IOC containing mock extracellular matrix, and “Maternal vessel” denotes the channel of the IOC containing endothelial cells, as described in the Methods section. Note the horizontal orientation of the actual devices. For purposes of data acquisition and interpretation, the IOC is imaged and shown in **(B, C)** in the vertical orientation, with the fetal channel at the top, ECM in the middle, and the maternal vessel channel at the bottom. **(B)** Brightfield imaging showing successful embedding of purified endometrial macrophages (Endo Mac), first trimester decidual macrophages (FT Mac), endometrial NK cells (Endo NK) and first trimester decidual NK cells (FT NK) onto the IOC. **(C)** Representative fluorescent imaging of labeled EVTs invading into the ECM and into the maternal vessel channel on the indicated post-seeding day. Days 1 and 3 post-seeding are shown. Channels on each device are separated by white dotted lines. After 1 day, note that most of the EVTs in any condition have not yet entered the ECM. After 3 days of culture, most of the EVTs are seen in the ECM channel in the macrophage and NK cell conditions. Scale bars represent 200μM. **(D)** Quantification of invasion area of EVTs beyond the fetal channel at day 3 of culture. One-way ANOVA with Tukey’s multiple comparison test was performed (n = 3 independent biological samples and 3 independent devices per biological sample). Data are presented as mean ± SD. Area of invasion at day 1 was not significantly different among groups. Invasion area of EVTs in the macrophage and NK cell conditions were all significantly different than the control (no immune cell) condition at day 3 of culture, ****, p<0.0001.

Since we saw significant gene expression differences between MSE and FT NK cells and macrophages, we next asked if we could identify functional differences in EVT invasion mediated by MSE and FT macrophages and NK cells using the IOC device (**Figure 7A**). We identified “bulk NK cells” as CD56+CD3- cells and “bulk macrophages” as CD14+CD64+ cells by flow cytometry and cell sorting. Recent work using flow cytometry, immunofluorescence, and transcriptional analyses supports that the vast majority of decidual-resident CD14+ cells are macrophages (39). To support that decidual CD14+CD64+ cells we purified for use on the IOC device are macrophages, we flow-sorted CD56+CD3- cells and CD14+CD64+ cells and prepared cytospins from FT decidua. As expected, decidual CD56+CD3- bulk NK cells exhibit lymphocytic morphology with abundant fine cytotoxic granules (**Supplementary Figure 6A**). Decidual bulk CD14+CD64+ cells appear similar to other morphologically large, vacuolated human macrophages in other contexts (**Supplementary Figure 6B**) (83).

We acknowledge that human myeloid subsets, particularly monocytes, macrophages, and DCs, share common ontogeny and have highly overlapping surface markers (84–86). In particular, tolerogenic DC10 cells have been show to express CD14, CD16, ILT4/LILRB2, HLA-G, DC-SIGN, CD163, and CD141/THBD on their surface. DC10 cells make IL-10 in the steady state and induce T regulatory cells. CD1c+ DC2s in the blood also contain CD14+ cells, and conventional flow cytometry is unable to completely distinguish CD14+ monocytes from DC2s (86). Only after unsupervised clustering using 92 protein markers could these DC2 cells be optimally distinguished from CD14+ monocytes. In our dataset, CD14, CD16 (FCGR3A), LILRB2, CD83, DCSIGN, CD163, CD141/THBD, and IL10 are all enriched in clusters predominantly identified as macrophages or monocytes (**Supplementary Figure 6C**). We observe minimal HLAG transcript among myeloid subclusters. Our data support that the vast majority of purified CD14+CD64+ cells used for IOC are macrophages.

We then incorporated 7000 bulk NK cells or bulk macrophages into the ECM of three IOC devices for each condition (**Figure 7A**). We quantified EVT invasion 1 and 3 days after cell seeding, when EVTs approached the EC channel. MSE and FT macrophages significantly enhanced invasion of EVTs (**Figures 7B–D**). We also recapitulated the pro-invasive effect we previously saw with MSE NK cells (41, 42) and found a similar increase in EVT invasion in the presence of FT decidual NK cells (**Figures 7B–D**). No differences were observed between the pro-invasive ability of macrophages and that of NK cells (**Figure 7D**).

The functional impact of maternal immune cells on fetal trophoblast cells is known to vary with gestational age, and we expected significant patient-patient variation. We therefore also examined our data in a more granular manner by replicate, since each replicate we carried out used EVTs (gestational age range: 7w3d - 7w5d) and maternal immune cells (FT gestational age range: 6w2d - 8w2d) from three different patients. When comparing the effects of MSE and FT macrophages, invaded area (**Supplementary Figure 6D**) in all replicates showed statistically significant increases in EVT invasion in the presence of macrophages at Day 3. Individual replicates of NK cells from MSE and FT samples also promoted significant increases in EVT invasion over control samples at Day 3. These data indicate that, similar to bulk NK cells, bulk macrophages also promote EVT invasion ex vivo on the IOC device. Additionally, despite many observed differentially expressed genes evident betweenMSE and FT macrophages and NK cells,pre- and post-implantation bulk macrophages, as well as pre- and post-implantation NK cells, function similarly ex vivo to promote EVT invasion on the IOC device.

## Discussion

Our data provide high-resolution insights into population dynamics and signaling events that shape the immune landscape of the pre- and post-implantation uterine lining. Using an implantation-on-a-chip device, we demonstrate that pre- and post-implantation innate immune cells can promote invasion of EVTs *ex vivo*. It is well established that NK cells promote invasion of fetal trophoblasts in numerous models, but conflicting reports exist regarding the effects of decidual macrophages on trophoblast invasion (87, 88). We show for the first time that bulk uterine CD14+CD64+ macrophages not only promote invasion of EVTs but do so as strongly as NK cells. Endometrial and post-implantation decidual bulk macrophages promoted EVT invasion to a similar degree *ex vivo*. While our data support that purified CD14+CD64+ cells are macrophages, additional work remains to be done to confirm the precise subsets of macrophages included and excluded by this gating strategy.

The precise factors and mechanisms responsible for the pro-invasive behavior of these cells in our reductionist *ex vivo* system, as well as in the more complex *in vivo* environment, remain to be determined. As the biology of the maternal-fetal interface is analogous to that of the tumor microenvironment in many ways, we found that the transcriptional profiles of macrophage subtypes we identified paralleled known subsets of tumor-associated macrophages. We found that each macrophage subtype was rich in transcripts encoding cytokines, chemokines, and growth factors that could plausibly promote EVT invasion or otherwise signal to fetal trophoblasts. AREG and EREG signal to EGFR, which has been shown in several studies to regulate normal and adverse outcomes of pregnancy, as has VEGF (89, 90). GPNMB promotes proliferation, invasion and metastasis of tumor cells (70). IL-1β promotes trophoblast migration *in vitro* (91). The contributions of each transcriptionally-distinct subset of macrophages (and NK cells) to implantation and placentation remain to be determined in studies ongoing in our labs.

Additional work is also ongoing to more deeply characterize the unique endometrial and decidual T subsets we observed in our prior (41) and current datasets. Regulatory DN T cells expressing cytolytic transcripts in endometrial tissue are emerging as intriguing players in the female reproductive system (92). DN T cells in our dataset were enriched in TIGIT transcript, which is also expressed at high levels in T-regulatory cells induced by regulatory macrophages (93). Further evidence is needed to establish precise functions of DN T cells during the window of implantation and during early pregnancy, when they may contribute to fetomaternal tolerance.

One limitation of these data is that they represent snapshots of immune populations in time and do not formally address the developmental trajectory of each immune subset. For instance, the overall increase in macrophages and decrease in monocytes in the first trimester decidua supports a number of different hypotheses. First, monocytes may differentiate into macrophages post-implantation. Second, monocytes may exhibit reduced trafficking to the decidua post-implantation, just as mouse monocytes traffic to the decidua only during early- to mid-gestation (72). Third, decidual macrophages may proliferate more or die less than decidual monocytes post-implantation.

Within the macrophage supercluster, we observed that M1a and M1b macrophages were relatively more abundant in the implantation-window endometrium. During the first trimester, M2 and M3 macrophages were more abundant than M1-subtypes in our cohort. This may reflect a need for more proangiogenic and proinflammatory macrophages during the peri-implantation period, as fetal trophoblasts invade into the decidua. After peak trophoblast invasion, as the definitive placenta develops, regulatory TAM-like M2 and M3 macrophages may be needed to lay the groundwork for immune tolerance thought to be essential for maintenance of pregnancy (94). Nevertheless, bulk endometrial macrophages/monocytes did not promote EVT invasion more strongly than bulk first trimester decidual macrophages/monocytes *ex vivo*. One explanation for this is that M1a and M1b macrophages may receive signals from trophoblasts in the post-implantation period to adopt a more M2- or M3-like transcriptome and phenotype, while still retaining their ability to attract EVTs. A better understanding of the microanatomic location of these macrophages in relation to trophoblasts is needed in order to contextualize their proinvasive function *in vivo.* Macrophages are often found in very close proximity to trophoblasts by imaging mass cytometry (40). Our data will allow us and others to refine the protein-level identification of transcriptionally-distinct macrophage subtypes, accelerating hypothesis-driven, mechanistic investigations into their functions *in vivo*. Future experiments aimed at understanding macrophage ontogeny and how decidual microenvironments shape the phenotype of macrophages during pregnancy are ongoing to address these outstanding questions.

Our data were generated from flow-sorted, cryopreserved CD45+ cells from dissociated uterine lining tissue. We thus compared scRNAseq of freshly isolated cells to flash-frozen-then-thawed cells from the same endometrial sample. While the sample size was limited, to our knowledge we are the first to rigorously assess the data quality of this approach in uterine tissue. Tissue dissociation and freezing is a practical way to bank precious human samples that may be available sporadically or at odd hours. We and others freeze dissociated samples to maximize the size of our tissue bank and to minimize batch effects introduced by individually processing and analyzing samples. The finding that freezing results in a modest underrepresentation of innate lymphocytes and, conversely, a modest overrepresentation of myeloid cells helps to guide comparison of our present and future data with studies using freshly isolated tissue. Similarly, modest changes in gene expression between fresh and frozen immune cells should also be taken into account when interpreting these and future data. Given our limited cell number in a single endometrial sample, additional work is needed to understand the differences between fresh and cryopreserved uterine immune populations. Our data suggest that subsets of ILCs may be more sensitive to cryopreservation and may be subject to more cell death upon thawing, compared to myeloid cells. However, the fact that our data closely align with prior studies using scRNAseq, flow/mass cytometry, and imaging mass cytometry (32, 33, 39, 40) lends extra validation to our approach to tissue processing.

By comparing the gene expression profiles of immune cells in the endometrium and first-trimester decidua, we uncovered gene signatures associated with the pre- and post-implantation periods. Across multiple subtypes of immune cells in the pre-implantation endometrium, we found enrichment of genes associated with proliferation, chemotaxis, and aerobic respiration. These data are consistent with the notion that, during decidualization in humans, the uterine lining recruits and supports tremendous amounts of immune cells that play critical roles in implantation and maintenance of pregnancy. One factor known to be required for implantation in mice is leukemia inhibitory factor (LIF), whose expression and activity peaks during decidualization in mice and humans (95–98). While luminal epithelial and stromal cells are thought to be the primary target of LIF, endometrial NK cells in our dataset exhibited enrichment of genes suggesting they also respond to LIF during the window of implantation. Indeed, antagonism of LIF signaling during mid-gestation is associated with abnormal spiral artery remodeling and placentation in mice (97). Future conditional deletion strategies of the LIF-LIFR axis will address the mechanisms by which this critically important molecule signals at the maternal-fetal interface.

In the post-implantation period, multiple macrophage and NK cell subsets were enriched for numerous IFN-stimulated genes. Type II IFN (IFNγ) derived from NK cells has long been known to be required for spiral artery remodeling in mice (99), but roles for Type I and type III IFNs in normal implantation and placentation have yet to be fully elucidated. It was shown recently that Type I IFN signaling inhibits trophoblast fusion to become syncytiotrophoblasts (100), helping to explain why fetal IFNAR mediates adverse outcomes of pregnancy in response to congenital Zika (101). Type III IFN has been shown to mediate placental defense against congenital viral infection (102). Our data support robust IFN tone after implantation and into the first trimester of normal pregnancy, raising questions about the specific IFNs produced and the mechanisms by which innate immune cells at the early maternal-fetal interface respond to IFNs to establish and maintain early pregnancy.

In summary, our data reveal that immune cells in the uterine lining undergo substantial changes after implantation of the embryo. Macrophages and NK cells, exhibit strong pro-invasive properties to guide early stages of placentation. As placentation must be carefully regulated to optimally nourish the developing fetus during pregnancy, these data lend further support to the essential role of innate immune cells awaiting arrival of the embryo. Our work will serve as a resource of future investigations into the mechanisms of how innate and adaptive immune cells communicate with fetal cells during the peri-implantation period and beyond.

## Data availability statement

The data presented in this study are deposited in the GEO database under accession number GSE255282. The code used to analyze these data are publicly accessible at: https://github.com/garifalloj/Scott-Gordon-Laboratory-CHOP-scRNAseq_Uterine_Macrophages_NK_Project.

## Ethics statement

The studies involving humans were approved by University of Pennsylvania Institutional Review Board. The studies were conducted in accordance with the local legislation and institutional requirements. The participants provided their written informed consent to participate in this study.

## Author contributions

SM: Conceptualization, Data curation, Formal analysis, Investigation, Methodology, Project administration, Resources, Software, Validation, Visualization, Writing – original draft, Writing – review & editing. JG: Data curation, Formal analysis, Investigation, Methodology, Software, Validation, Visualization, Writing – review & editing. SK: Data curation, Formal analysis, Investigation, Methodology, Software, Validation, Visualization, Writing – review & editing. MS: Data curation, Formal analysis, Investigation, Methodology, Software, Visualization, Writing – review & editing. DH: Data curation, Formal analysis, Funding acquisition, Investigation, Methodology, Project administration, Resources, Software, Supervision, Visualization, Writing – review & editing. SG: Conceptualization, Data curation, Formal analysis, Funding acquisition, Investigation, Methodology, Project administration, Resources, Software, Supervision, Validation, Visualization, Writing – original draft, Writing – review & editing. MM: Conceptualization, Data curation, Formal analysis, Funding acquisition, Investigation, Methodology, Project administration, Resources, Software, Supervision, Validation, Visualization, Writing – original draft, Writing – review & editing.
