# Supplementary material for: Uterine macrophages and NK cells exhibit population and gene-level changes after implantation but maintain pro-invasive properties
Source: Front Immunol. 2024 Mar 19;15:1364036. doi: 10.3389/fimmu.2024.1364036 (PMC10985329; doi:10.3389/fimmu.2024.1364036)
Supplement: Supplementary file 1 [file DataSheet_1.pdf]

Supplementary Material

Uterine macrophages and NK cells exhibit population- and gene-level changes after implantation but maintain pro-invasive properties

Sneha Mani,[1] James Garifallou,[2] Se-jeong Kim,[3] Michael K. Simoni,[1] Dan Dongeun Huh,[3,4] Scott M. Gordon,[2,5,]* Monica Mainigi[1,]*

[1]Division of Reproductive Endocrinology and Infertility, University of Pennsylvania, Perelman School of Medicine, Philadelphia, PA, USA

[2]Division of Neonatology, Children's Hospital of Philadelphia, Philadelphia, PA, USA

[3]Department of Bioengineering, School of Engineering and Applied Science, University of Pennsylvania, Philadelphia, PA, USA

[4]Center for Engineering MechanoBiology, School of Engineering and Applied Science, University of Pennsylvania, Philadelphia, PA, USA

[5]Department of Pediatrics, University of Pennsylvania, Perelman School of Medicine, Philadelphia, PA, USA

* These authors contributed equally to this work and share senior authorship, listed in alphabetical and ascending height order.

Correspondence: gordons1@chop.edu, monica.mainigi@pennmedicine.upenn.edu

SUPPLEMENTARY FIGURE 1

A

Condition	Patient	Ethnicity	Age	BMI		Prior Losses
MSE/ENDO	1	Asian	23	19.8	-	0
MSE/ENDO	2	White	29	20.9	-	0
MSE/ENDO	3	White	33	23.8	-	0
MSE/ENDO	4	White	23	24	-	0

Condition	Patient	Ethnicity	Age	BMI	EGA	Prior Losses
FT	1	White	32	20.3	5w6d	0
FT	2	African-American	29	23.1	6w3d	0
FT	3	African-American	29	28.5	9w3d	0
FT	4	African-American	24	29.1	11w4d	1

B

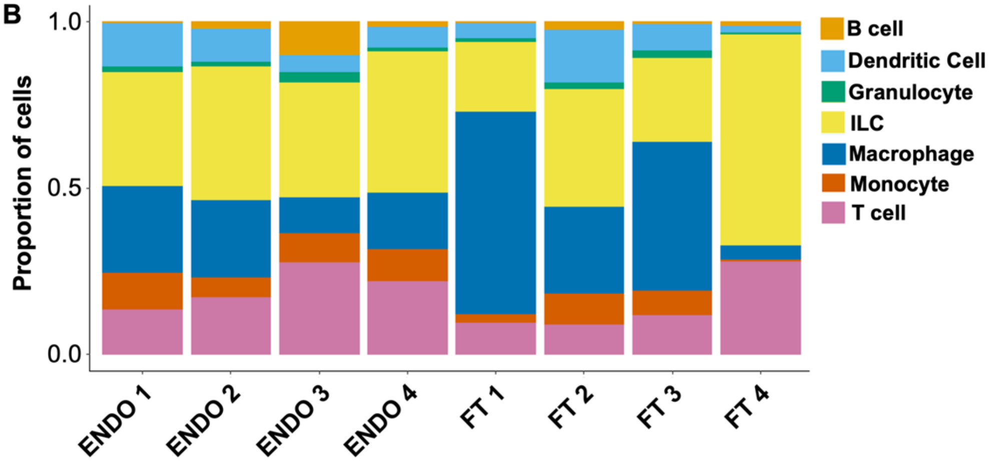

C

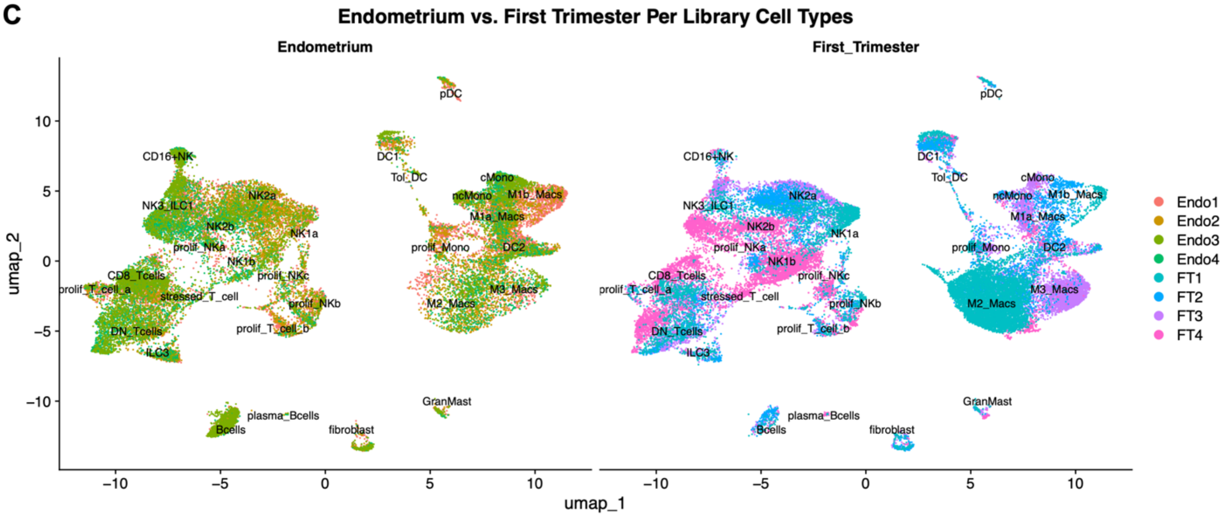

Supplementary Figure 1. Demographics of patients included in this study and immune composition by supercluster of individual samples.

(A) Demographics of subjects from whom CD45+ cells were obtained for scRNAseq. MSE/ENDO, window of implantation (mid-secretory) endometrium. FT, first trimester decidua.

(B) Stacked bar graphs show each supercluster as a percentage of total CD45+ cells in indicated samples of window of implantation (mid-secretory) endometrium (ENDO) and first trimester decidua (FT).

(C) Split UMAPs show the contributions of individual samples to endometrial (Endo) and first trimester (FT) decidual subclusters.

SUPPLEMENTARY FIGURE 2

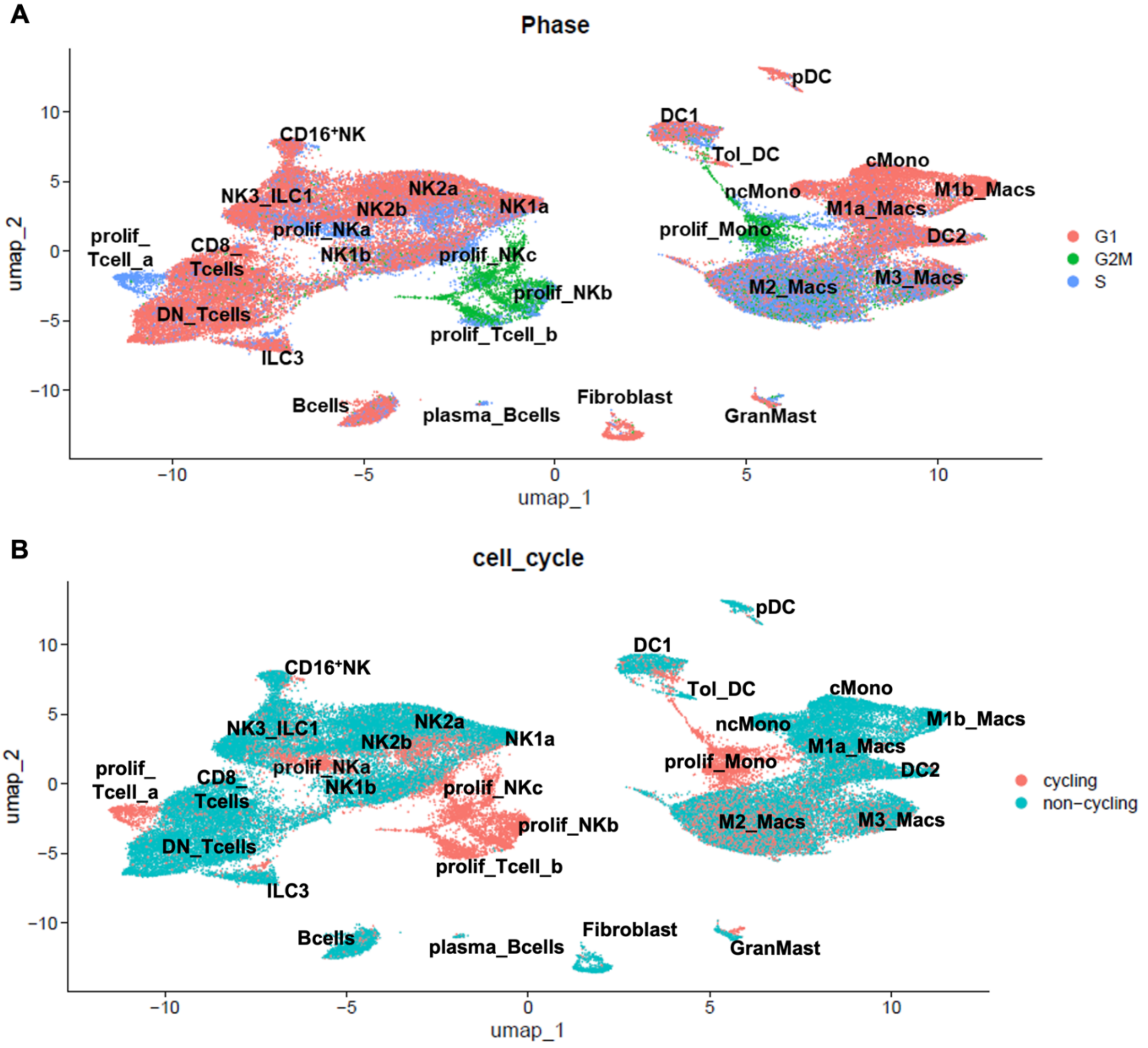

Supplementary Figure 2. Cell cycle analysis guides identification of proliferative immune subclusters.

(A) Cells shown in red are predicted to be in the G1 phase of the cell cycle, cells shown in green are predicted to be in G2/M phase, and cells shown in blue are predicted to be in S phase.

(B) Similar to (A), cells shown in red are predicted to have entered the phases of the cell cycle associated with active division, while teal cells are not predicted to be actively dividing.

SUPPLEMENTARY FIGURE 3

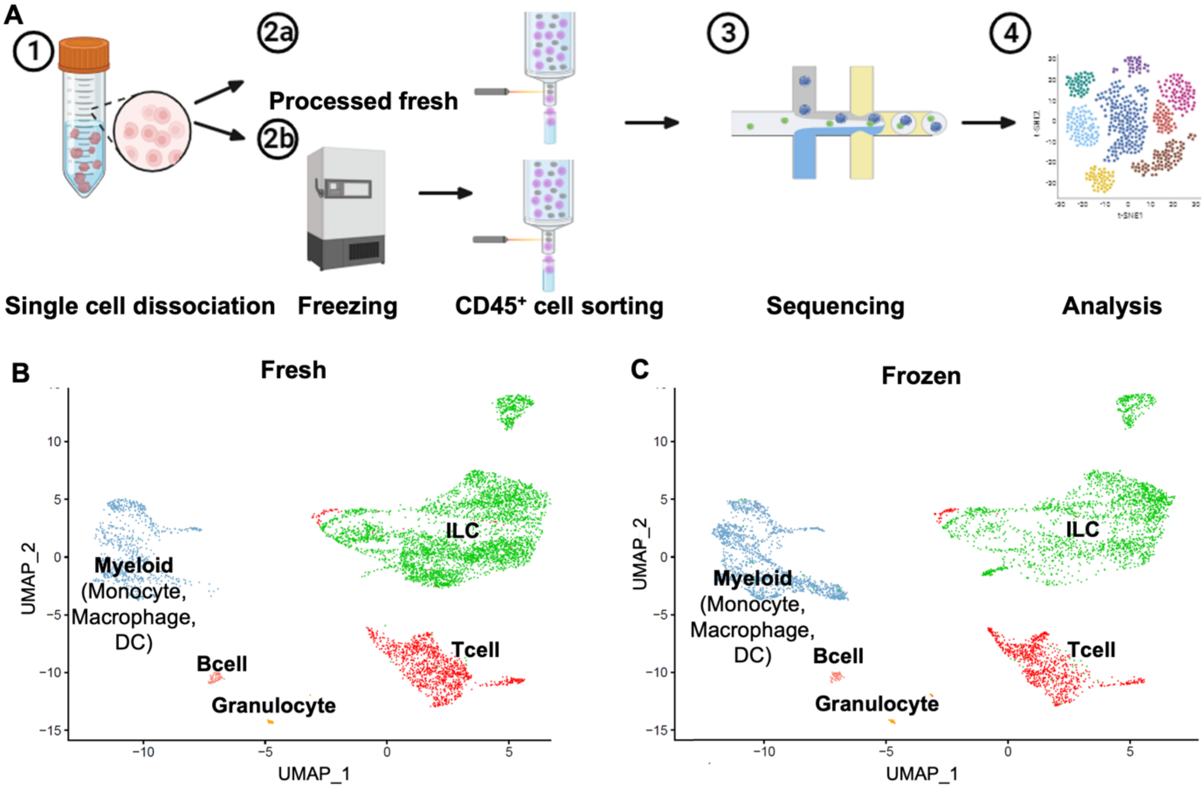

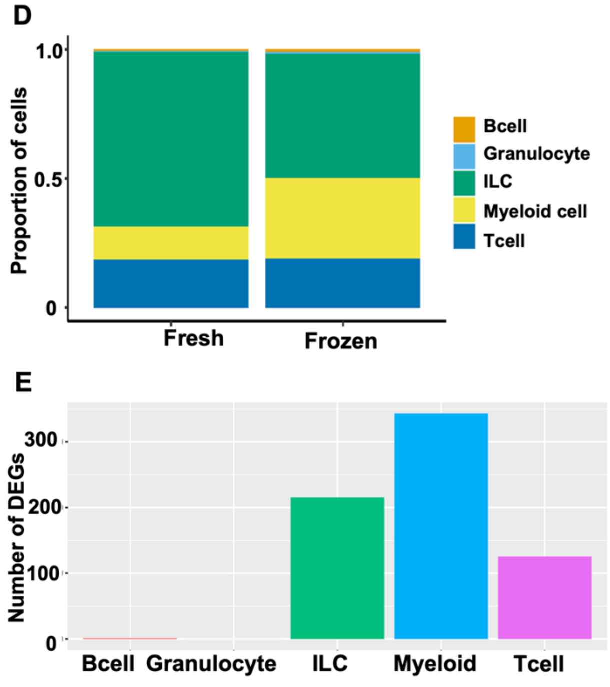

F

Myeloid

GENE SYMBOL	AVG LOG2FC	ADJ P VALUE
GNLY	2.773140706	0.000100081
NKG7	1.560181824	1.84E-05
GZMA	1.439200012	3.05E-05
IL2RB	1.388372409	4.41E-10
TRDC	1.077737589	8.44E-05
KLRB1	1.00135502	0.0001023
IL32	0.778293254	0.033197277
RPS10	0.668243728	5.65E-77
LGALS2	0.582725433	2.96E-18
CXCR4	0.565182655	0.001314232
CD300E	0.556975733	0.000298315
FCER1A	0.491461162	0.001236627
RPL23	0.472650995	4.02E-40
AREG	0.470100372	1.53E-06
RPS11	0.45288378	7.28E-53
CD1C	0.452081876	0.001355567
RPS20	0.444437081	3.08E-20
RPL7	0.433984623	2.30E-43
RPL31	0.429344851	1.15E-27
FOSL2	0.423073931	3.57E-05
IGF1	-1.39469571	1.88E-15
MSR1	-1.42313905	3.44E-23
FCGBP	-1.45020023	4.11E-18
SLCO2B1	-1.46293799	3.18E-23
A2M	-1.62195104	5.05E-21
STAB1	-1.6320888	3.72E-24
GAL3ST4	-1.67721967	6.59E-27
LYVE1	-1.72912978	1.39E-14
APOC1	-1.76372745	8.74E-20
MAF	-1.82712141	4.80E-24
PLTP	-1.84705175	6.13E-28
HAMP	-1.88080831	4.38E-19
FOLR2	-2.12629776	9.46E-36
DAB2	-2.17925705	3.68E-21
CCL2	-2.22074164	9.45E-20
NRP1	-2.38230962	2.72E-28
APOE	-2.48653915	6.88E-31
SPP1	-2.66783398	9.56E-06
SELENOP	-2.83889601	2.74E-27
RNASE1	-3.7120661	7.34E-24

ILCs

GENE SYMBOL	AVG LOG2FC	ADJ P VALUE
CYP26A1	1.182911436	9.08E-41
SPINK2	1.068238959	1.16E-23
GNLY	1.058311845	6.12E-94
IGFBP7	1.056597177	2.09E-47
KIR2DL3	0.971549397	2.79E-47
ADAMTS1	0.919063879	2.96E-28
AFAP1L2	0.882989684	1.17E-44
ITGAD	0.84760035	2.98E-45
SLC12A8	0.833331168	2.42E-31
CSF1	0.830772466	2.72E-25
KIR2DL1	0.801509892	7.51E-23
EPAS1	0.784828344	2.65E-29
IL2RB	0.752076257	5.40E-149
MYO1E	0.715569388	1.33E-25
SLC44A1	0.710101712	5.49E-27
ACY3	0.702408294	7.00E-17
GZMB	0.699019509	1.02E-58
KIR3DL1	0.697150963	6.05E-17
ABHD2	0.631028183	3.51E-28
HIPK2	0.613581866	1.09E-25
AC253572.2	-0.59914772	7.46E-11
MTRNR2L8	-0.6021063	5.84E-08
PFN1	-0.61602268	4.52E-161
MT-ATP8	-0.62418172	2.11E-44
TUBA1A	-0.64166352	3.79E-37
SEPTIN1	-0.65149936	1.13E-34
ITM2C	-0.65716653	1.47E-24
IFITM1	-0.69523371	2.83E-24
ALOX5AP	-0.71741684	1.47E-63
RGS2	-0.71891451	1.02E-27
LTB	-0.73504358	4.38E-23
LINC01871	-0.75624479	1.40E-25
ARF5	-0.80881552	1.96E-35
LYZ	-1.14345605	4.20E-68
CST3	-1.16993438	1.64E-85
HLA-DPB1	-1.23909116	1.21E-80
CD74	-1.28848108	2.37E-151
HLA-DPA1	-1.33397876	2.59E-107
HLA-DRB1	-1.76235973	1.66E-190
HLA-DRA	-1.89840892	1.98E-216

Increased in Fresh

Increased in Frozen

Supplementary Figure 3. Similar clustering, similar gene expression profiles, and modest relative decrease in ILC representation when sequencing previously frozen uterine immune cells.

(A) Graphical representation of workflow to dissociate MSE tissue, process and sort CD45+ cells fresh or frozen/thawed, and perform scRNAseq.

(B, C) UMAP plots showing superclustering of CD45+ cells meeting quality metrics, recovered from fresh (B) and frozen/thawed (C) MSE. ILC, innate lymphoid cells.

(D) Stacked bar charts showing proportion of total CD45+ cells represented by each supercluster in fresh and frozen/thawed MSE.

(E) Bar graph showing absolute number of significant, differentially expressed genes (DEGs) in indicated fresh versus frozen/thawed superclusters. Significant genes are defined as having an adjusted p value of <0.05 and an absolute fold change of 25% or greater (either ≥25% enriched in fresh or ≥25% in frozen).

(F) List of the top 20 DEGs enriched in fresh (highlighted in pale red) or frozen (highlighted in pale yellow) myeloid cells (left) and ILCs (right). Official gene symbol, average log2 fold change, and adjusted p values are shown.

SUPPLEMENTARY FIGURE 4

Macrophages

M1a

GENE SYMBOL	AVG LOG2FC	ADJ P VALUE
SPP1	-4.638317418	2.98E-240
APOE	-3.422933287	3.13E-125
RNASE1	-2.942427864	2.10E-134
PLIN2	-2.435063017	7.52E-107
IFIT2	-2.315354314	1.75E-75
CTSL	-2.273440363	8.15E-125
MSR1	-2.266002664	1.60E-153
IFIT3	-2.165358975	9.49E-109
ISG15	-2.144800948	1.40E-92
IFIT1	-2.070112891	1.32E-77
FABP5	-2.053047327	4.33E-65
APOC1	-2.04401613	1.58E-22
FN1	-1.911606611	3.07E-16
IFITM3	-1.774934547	2.90E-162
C15orf48	-1.739616828	3.29E-118
IFI6	-1.721137884	3.52E-100
MX1	-1.685606731	8.60E-90
C1QC	-1.520482531	1.98E-68
GPNMB	-1.517720264	2.45E-34
IFI44L	-1.507481269	1.81E-80
SELENOK	1.115505488	1.96E-56
MEGF9	1.122443393	3.32E-42
CYCS	1.13597871	1.05E-59
OSM	1.220625209	1.14E-32
RGS2	1.22407144	2.18E-118
LTA4H	1.255422013	2.00E-90
HBEGF	1.269804034	7.06E-92
S100A9	1.282570643	1.81E-93
FCN1	1.290366926	3.23E-187
CD1D	1.329404422	8.40E-76
HSPA1B	1.340120503	1.69E-82
HLA-DRB5	1.346718089	8.57E-19
S100A8	1.523703371	1.41E-97
FBP1	1.611252697	3.54E-124
ALOX5AP	1.67504248	3.89E-131
CD52	1.768854927	6.33E-169
MCEMP1	1.916024607	8.06E-84
LYZ	1.976816344	0
PLAC8	2.216325805	7.42E-148
RETN	2.750379612	1.08E-112

M1b

GENE SYMBOL	AVG LOG2FC	ADJ P VALUE
SELENOP	-3.4415097	2.77E-49
HLA-DRB5	-3.243605499	5.73E-66
RNASE1	-3.046674328	3.60E-45
C1QC	-2.331358802	5.53E-39
C1QB	-2.212345034	8.04E-33
C3	-2.176899447	6.28E-29
AC079753.2	-2.103720904	5.92E-49
C1QA	-1.94455187	1.75E-25
OTUD1	-1.517991706	2.93E-26
GBP2	-1.25724121	1.70E-22
TMEM176B	-1.251622022	3.85E-20
TNFAIP8	-1.16969046	4.94E-13
PLCG2	-1.154105498	1.46E-41
TNF	-1.151543835	1.18E-26
KCNJ2	-1.12453467	2.15E-09
AC253572.2	-1.106470433	2.95E-11
AC016831.1	-1.106252747	2.78E-10
PELI1	-1.066061856	3.83E-09
CCL4L2	-1.030213141	5.46E-15
TMEM176A	-0.979282339	3.16E-10
IFI30	1.014130679	3.31E-29
ARL4A	1.072553529	7.75E-07
AL034397.3	1.072708793	6.69E-06
CAPG	1.089375807	5.07E-15
ATP1B3	1.101648473	4.71E-15
RNASE2	1.14674881	4.77E-05
LGALS1	1.158075546	2.45E-41
SELENOK	1.17034108	5.93E-12
TIMP1	1.301102966	2.51E-32
SMIM25	1.319207694	9.97E-16
RPS26	1.319979273	2.73E-51
ALOX5AP	1.339827513	2.94E-06
MARCO	1.37225202	5.63E-08
LYZ	1.372532812	9.79E-88
TWISTNB	1.609231467	1.93E-05
PLAC8	1.690365705	2.14E-12
HBEGF	2.043722294	5.39E-35
MCEMP1	2.081796817	9.00E-23
FBP1	2.348415738	6.54E-19
RETN	2.976923464	5.06E-14

M2

GENE SYMBOL	AVG LOG2FC	ADJ P VALUE
SELENOP	-2.439964321	0
IFI44L	-2.183662157	3.29E-174
MTSS1	-2.092628258	2.20E-219
JUN	-2.033339645	0
CXCL12	-1.978001633	1.37E-113
EGR1	-1.88574926	1.00E-206
PLTP	-1.677683422	5.27E-204
RNASE1	-1.648905398	2.15E-294
SERPING1	-1.62588744	2.88E-116
GYPC	-1.556851464	7.72E-194
C3	-1.453697847	5.64E-205
F13A1	-1.450850343	2.74E-229
IGF1	-1.446924203	6.08E-164
TNFAIP3	-1.446276681	3.52E-117
DUSP6	-1.427105908	8.84E-187
SIGLEC1	-1.402423088	2.20E-116
KLF4	-1.37306504	3.78E-148
AC020916.1	-1.372529963	4.49E-170
FOSB	-1.366707814	9.40E-260
AZIN1-AS1	-1.338123165	3.09E-43
RPS26	1.245287923	1.40E-231
SH3BGRL3	1.255977852	4.56E-270
LST1	1.293226532	7.20E-288
CKLF	1.298491742	4.89E-130
FABP5	1.305355244	4.42E-127
CAMK1	1.314733217	2.17E-159
OLR1	1.321581445	5.52E-125
CD48	1.332224142	5.23E-133
IFI30	1.367408348	0
CAPG	1.392313109	9.39E-251
PKIB	1.494995422	2.49E-160
LSP1	1.512817154	2.63E-180
S100A6	1.583122281	9.50E-163
TIMP1	1.61011874	3.61E-187
S100A4	1.684824959	9.15E-264
APOC2	1.851674482	9.06E-59
PPA1	1.866487525	1.74E-283
LGALS1	2.015593415	0
CLEC10A	2.201682858	1.07E-292
LYZ	3.180809276	0

M3

GENE SYMBOL	AVG LOG2FC	ADJ P VALUE
SPP1	-3.09564238	7.32E-139
RNASE1	-3.0524065	4.45E-174
CD28	-2.342372045	6.96E-62
SELENOP	-2.220786186	8.43E-108
IFI44L	-2.125124548	6.47E-60
AC253572.2	-2.124581801	3.73E-34
MSR1	-2.110509923	4.34E-130
PLTP	-2.091330361	9.91E-92
MX1	-2.008745289	1.25E-70
IFIT2	-1.997888471	1.95E-27
TMIGD3	-1.991682969	6.79E-29
SDC2	-1.989267739	2.24E-35
FABP5	-1.84693005	1.44E-52
IFIT3	-1.817953622	3.83E-37
AL021155.5	-1.813318542	1.35E-20
AC007952.4	-1.798364341	6.22E-42
C2	-1.796847478	1.23E-65
ITSN1	-1.757379875	7.62E-51
IFIT1	-1.750805244	5.89E-30
CADM1	-1.725718181	3.99E-33
ALDH2	1.408923465	2.68E-50
FGL2	1.426427944	4.71E-80
WARS	1.429078129	5.50E-36
RGS2	1.493943124	2.11E-42
JAML	1.504359769	1.33E-49
AC020656.1	1.559001279	1.56E-39
CD52	1.586117229	5.08E-43
LST1	1.611174948	5.19E-106
ALOX5AP	1.687785984	1.48E-68
IL1B	1.701500487	1.71E-16
HLA-DQB1	1.919486116	3.43E-162
LTA4H	1.936691276	1.04E-62
CYP27A1	2.16774256	6.45E-79
GPR183	2.217896581	1.56E-112
OLR1	2.248277274	4.93E-94
HLA-DQA1	2.521097506	1.15E-213
CLEC10A	2.562361323	1.26E-173
HLA-DRB5	2.675710706	2.99E-45
LYZ	3.234862239	2.61E-161
FCN1	3.347605528	3.60E-144

Increased in FT

Increased in Endo

Supplementary Figure 4. Top genes differentially expressed between endometrial and first trimester decidual macrophages.

List of the top 20 DEGs enriched in first trimester decidual (highlighted in pale blue) or pre-implantation endometrial (white cells, unhighlighted) macrophage subclusters. Official gene symbol, average log2 fold change, and adjusted p values are shown.

SUPPLEMENTARY FIGURE 5

ILCs

Increased in FT (rows 1–20 of each table, highlighted); Increased in Endo (rows 21–40 of each table)

NK1a

GENE SYMBOL	AVG LOG2FC	ADJ P VALUE
SELENOP	-3.286487378	5.13E-70
RNASE1	-3.082556211	4.46E-61
APOE	-2.811170483	2.65E-47
C1QB	-2.483396056	6.89E-94
C1QA	-2.465858114	1.86E-97
C1QC	-2.313473019	1.37E-72
CD14	-1.946570705	1.04E-52
SPTSSB	-1.531807209	8.40E-29
CTSB	-1.512068103	8.61E-37
JUN	-1.47534051	6.80E-34
ENTPD1	-1.465226928	5.19E-31
CD74	-1.386473001	6.75E-49
CDHR1	-1.362644826	2.80E-13
FTL	-1.347393971	3.60E-89
GRN	-1.297783985	9.27E-23
TTN	-1.26245793	0.006650051
AC020916.1	-1.218361039	9.09E-07
AL627171.2	-1.216333816	8.70E-34
SPINK2	-1.175458988	1.67E-06
AREG	-1.128427028	0.768305101
H2AFZ	0.972416938	7.95E-35
MCM7	0.999571253	2.54E-24
PCNA	1.063885837	5.13E-25
RGCC	1.068351157	6.11E-27
HSPE1	1.086633619	8.01E-53
TUBB	1.088009826	1.72E-28
CRIP1	1.094339067	7.31E-71
LGALS1	1.103660131	5.19E-59
CCL4	1.134483705	4.00E-58
MCM6	1.141489496	1.47E-31
MCM3	1.156073113	8.26E-29
HELLS	1.169755773	7.42E-31
STMN1	1.20292107	1.67E-32
KRT81	1.217706336	6.89E-40
MT2A	1.329581252	1.48E-34
KRT86	1.332648024	1.55E-42
XCL2	1.378559642	1.55E-72
XCL1	1.878432772	5.49E-99
CD9	1.893770747	3.67E-88
LYZ	1.897424676	2.34E-39

NK1b

GENE SYMBOL	AVG LOG2FC	ADJ P VALUE
IGKC	-3.38274942	3.38E-134
IFI44L	-2.74549926	3.20E-56
ERAP2	-1.75157023	1.84E-52
C1QA	-1.51257796	6.33E-41
GOLGA8O	-1.48837148	1.47E-29
MT-ND4L	-1.48494501	6.68E-160
MTRNR2L8	-1.39460687	2.39E-50
SLFN5	-1.29036196	7.39E-83
IL32	-1.284252	7.40E-79
LINC00426	-1.28308916	4.87E-20
CD2	-1.26964224	5.36E-40
XAF1	-1.24371811	1.16E-31
MT-ATP8	-1.23300606	1.76E-93
SLC44A1	-1.22749697	4.22E-27
POLR2J3	-1.21289231	4.04E-35
C1QB	-1.16784134	3.00E-27
BCL2	-1.1576661	5.37E-20
SNHG14	-1.14450564	1.65E-16
ABCA2	-1.09315197	4.50E-19
DTHD1	-1.08233797	1.14E-22
ZNF683	1.46348116	5.54E-41
CRIP1	1.46352185	1.77E-102
FOSB	1.496860774	3.34E-62
VPS37B	1.497263836	7.00E-53
REL	1.551583527	9.21E-65
CD52	1.554015097	5.49E-109
FOSL2	1.619735028	1.22E-67
DNAJB1	1.651133531	9.87E-55
LDLRAD4	1.737624112	8.68E-54
RASGEF1B	1.752732865	9.83E-63
AC020916.1	1.758993431	1.89E-72
CD69	1.882197153	4.51E-87
TNFAIP3	2.07018311	4.96E-82
RGS1	2.105997426	3.38E-111
CCL4	2.112263296	3.92E-176
NR4A1	2.167304966	1.38E-95
ZNF331	2.335125629	3.82E-92
LYZ	2.444041285	1.74E-198
DUSP2	2.492049672	2.74E-139
NR4A2	3.4433553	1.57E-298

NK2a

GENE SYMBOL	AVG LOG2FC	ADJ P VALUE
AREG	-2.279868729	6.91E-240
TSC22D3	-1.762126651	3.20E-211
APOC2	-1.521658905	7.28E-83
FTL	-1.213380877	3.38E-211
SOCS1	-1.211820709	1.20E-121
CDHR1	-1.180227896	3.63E-70
DDIT4	-1.070720681	1.79E-108
JUN	-1.03059738	4.62E-16
ZFP36L2	-1.027237395	1.69E-192
TXNIP	-1.021000935	5.30E-69
DUSP1	-0.956729478	3.00E-58
CD74	-0.920117619	8.46E-84
CEBPD	-0.85906764	2.52E-35
JUNB	-0.847355756	2.75E-50
ZFP36	-0.840346381	2.50E-76
PNRC1	-0.81463365	5.95E-76
IFITM1	-0.759141732	1.96E-15
TTN	-0.75374794	1.04E-17
IGFBP7	-0.731554468	3.10E-32
ID2	-0.710936116	1.33E-78
CD247	0.703307808	2.97E-52
DUT	0.707683957	2.43E-16
TUBB	0.720117146	2.24E-29
NME1	0.725185111	1.05E-29
RGCC	0.729395112	1.24E-43
KRT81	0.733557734	2.83E-75
STMN1	0.740151364	2.86E-20
SRM	0.747474673	2.49E-31
DCTPP1	0.75578065	1.81E-34
CXXC5	0.761034302	4.71E-30
IFI27L2	0.764337886	1.39E-43
CRIP1	0.796084557	1.85E-117
RGS16	0.825089379	4.06E-22
TNFRSF18	0.837062373	1.46E-101
KRT86	0.914678537	1.01E-86
FASLG	1.018460434	5.37E-68
CYBA	1.050259811	2.20E-85
CD52	1.170277439	3.33E-193
CD9	1.248095843	5.88E-112
LTB	1.435621191	5.99E-68

NK2b

GENE SYMBOL	AVG LOG2FC	ADJ P VALUE
IGKC	-2.76513771	1.16E-92
IGFBP7	-2.1002317	1.12E-49
IFITM1	-1.86353654	2.04E-82
ERAP2	-1.68379408	3.22E-35
SLC44A1	-1.57205007	2.18E-31
CMC1	-1.47057453	3.32E-73
CD2	-1.46758877	3.60E-74
ISG20	-1.44961106	5.14E-33
ITGAD	-1.43657197	3.53E-31
PLCG2	-1.40772035	3.13E-155
MX1	-1.40755471	1.01E-20
APOC2	-1.35097484	3.96E-18
MT-ATP8	-1.33955565	1.01E-69
GZMB	-1.29668484	4.10E-46
IFITM3	-1.28376097	5.02E-27
MT-ND4L	-1.26651283	3.43E-85
XAF1	-1.2592379	9.39E-25
GBP5	-1.12039969	2.03E-15
AFAP1L2	-1.11894635	1.12E-14
ISG15	-1.06081309	1.37E-21
BTG2	1.421672573	1.32E-31
LTB	1.446373815	6.84E-20
ALOX5AP	1.496245685	3.21E-35
RGCC	1.555652971	7.85E-32
FOSB	1.68133614	2.27E-35
DUSP1	1.743210527	4.62E-29
CCL3	1.837317794	1.35E-26
TNFAIP3	1.871572495	7.55E-48
ZNF331	1.891877686	2.59E-37
JUN	1.961590153	6.11E-41
CD160	2.030034669	1.11E-30
RGS1	2.052482398	2.12E-55
FOS	2.074518818	8.73E-70
JUNB	2.148435508	2.32E-55
CCL4L2	2.175643493	3.07E-24
CD69	2.257855946	2.78E-91
CCL4	2.271248312	3.70E-108
CXCR4	2.423474534	1.06E-54
CD9	2.661673804	1.07E-76
DUSP2	2.686693559	6.03E-100

NK3/ILC1

GENE SYMBOL	AVG LOG2FC	ADJ P VALUE
IGKC	-2.32510205	1.36E-171
ISG20	-1.91911875	2.65E-145
IFITM1	-1.63162994	7.61E-132
TSC22D3	-1.41498207	2.17E-39
CMC1	-1.34506182	1.71E-125
MT2A	-1.34032774	6.15E-70
ISG15	-1.27872845	7.17E-68
STAT1	-1.19715075	1.96E-43
XAF1	-1.14773989	1.21E-45
IFITM3	-1.10027929	7.98E-36
CD2	-1.08141106	1.09E-43
PLCG2	-1.05224044	1.27E-139
EPSTI1	-0.97696566	2.58E-33
CEBPD	-0.95331126	1.27E-45
GBP5	-0.94104852	1.16E-27
GZMK	-0.92335229	1.05E-32
SLFN5	-0.89997747	1.44E-58
RPS26	-0.83310219	2.41E-127
LYST	-0.80538633	1.10E-21
CD74	-0.77972827	7.33E-48
MAP3K8	0.860412769	4.02E-29
SDF2L1	0.862016341	1.44E-23
ITGA1	0.866028079	3.71E-31
MANF	0.906615418	8.56E-26
ZNF331	0.909762846	6.26E-40
CAPG	0.923168201	2.17E-48
CCL4	0.979949737	4.30E-161
JUND	1.03444784	3.39E-186
FASLG	1.050403673	9.39E-39
EZR	1.055925139	1.33E-52
ITM2C	1.057525532	4.31E-68
LDLRAD4	1.14215048	1.03E-38
IGFBP2	1.156271131	2.23E-74
NR4A2	1.175737921	9.53E-82
TNFRSF18	1.183732138	4.72E-68
CD52	1.442583377	3.35E-96
KRT86	1.519965014	7.37E-130
KRT81	1.628185489	1.26E-141
LYZ	1.766641967	1.11E-75
CD9	2.09086451	3.08E-135

Supplementary Figure 5. Top genes differentially expressed between endometrial and first trimester decidual ILCs.

List of the top 20 DEGs enriched in first trimester decidual (highlighted in pale blue) or pre-implantation endometrial (white cells, unhighlighted) ILC subclusters. Official gene symbol, average log2 fold change, and adjusted p values are shown.

SUPPLEMENTARY FIGURE 6

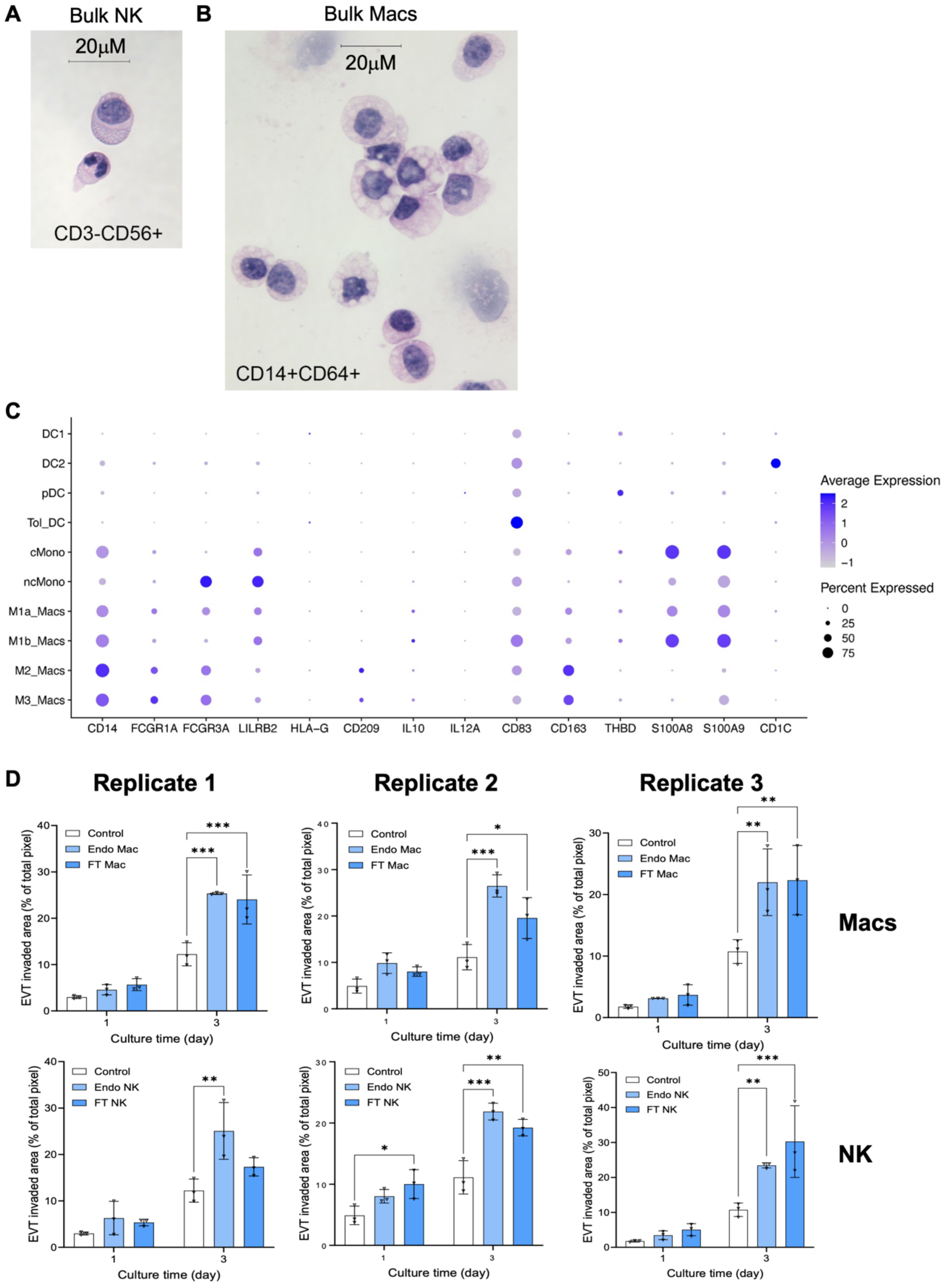

Supplementary Figure 6. Pre- and post-implantation bulk CD14+CD64+ macrophages promote invasion of primary EVTs as strongly as pre- and post-implantation bulk NK cells ex vivo on an implantation-on-a-chip device.

(A,B) H&E-stained cytospins were prepared from cryopreserved first-trimester decidual flow-sorted cells and imaged with a Leica brightfield microscope at 63x. The scale bar shown is 20 microns. Shown are (A) CD3-CD56+ "Bulk NK cells" that appear lymphocytic with abundant fine cytotoxic granules. In (B), CD14+CD64+ "Bulk Macrophages" are predominantly large, heavily vacuolated cells that resemble tissue macrophages.

(C) Dot plot representation of DC10-associated genes expressed among indicated myeloid subclusters.

(D) Quantification of individual biological replicates on the implantation-on-a-chip (IOC). Three technical replicates per biological replicate are shown. Invasion area of EVTs beyond the fetal channel at days 1 and 3 of culture are shown. One-way ANOVA with Tukey's multiple comparison test was performed. Data are presented as mean ± SD. *, $p<0.05$; **, $p<0.01$; ***, $p<0.001$.
